# Structure Evolution of Ge-Doped CaTiO_3_ (CTG) at High Pressure: Search for the First 2:4 Locked-Tilt Perovskite by Synchrotron X-ray Diffraction and DFT Calculations

**DOI:** 10.1021/acs.inorgchem.3c02645

**Published:** 2023-10-05

**Authors:** Matteo Ardit, Sonia Conte, Donato Belmonte, Francesca Menescardi, Simone Pollastri, Giuseppe Cruciani, Michele Dondi

**Affiliations:** † Department of Physics and Earth Sciences, University of Ferrara, via Saragat 1, I-44122 Ferrara, Italy; ‡ CNR-ISSMC, Institute of Science, Technology and Sustainability for the Development of Ceramic Materials, via Granarolo 64, I-48018 Faenza, Italy; § Department of Earth Sciences, Environment and Life (DISTAV), University of Genova, corso Europa 26, I-16132 Genova, Italy; ∥ ELETTRA - Sincrotrone Trieste, ss 14, km 163.5, I-34149 Basovizza, Italy

## Abstract

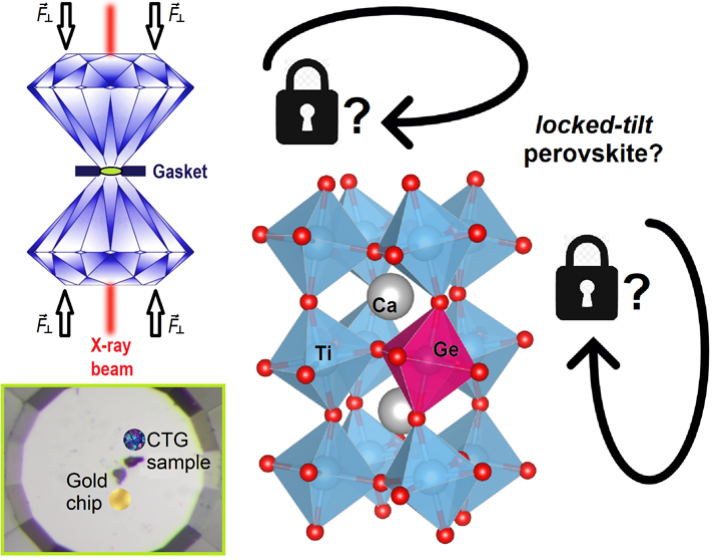

This research investigates the high-pressure behavior of the Ca(Ti_0.95_Ge_0.05_)O_3_ perovskite, a candidate of the locked-tilt perovskite family (orthorhombic compounds characterized by the absence of changes in the octahedral tilt and volume reduction under pressure controlled solely by isotropic compression). The study combines experimental high-pressure synchrotron diffraction data with density functional theory (DFT) calculations, complemented by the X-ray absorption near-edge structure (XANES) and extended X-ray absorption fine structure (EXAFS), to understand the structural evolution of the perovskite under pressure. The results show that CTG undergoes nearly isotropic compression with the same compressibility along all three unit-cell axes (i.e., *K*_*a*0_ = *K*_*b*0_ = *K*_*c*0_, giving a normalized cell distortion factor with pressure *d*_norm_(*P*) = 1). However, a modest increase in octahedral tilting with pressure is revealed by DFT calculations, qualifying CTG as a new type of GdFeO_3_-type perovskite that exhibits both isotropic compression and nonlocked tilting. This finding complements two existing types: perovskites with anisotropic compression and tilting changes and those with isotropic compression and locked tilting. The multimethod approach provides valuable insights into the structural evolution of locked-tilt perovskites under high pressure and establishes a protocol for the efficient study of complex high-pressure systems. The results have implications for the design of new functional materials with desirable properties.

## Abstract 2

This study investigates the high-pressure behavior of the Ca(Ti_0.95_Ge_0.05_)O_3_ perovskite (CTG), a candidate locked-tilt perovskite. Combining experimental analysis and DFT calculations, the study shows that CTG undergoes nearly isotropic compression and exhibits a modest increase in octahedral tilting under pressure, making it a unique GdFeO_3_-type perovskite. The results provide valuable insights into the behavior of perovskites and their potential for the design of new functional materials.

## Introduction

Recent high-pressure synchrotron structural studies have identified YAl_0.25_Cr_0.75_O_3_, a member of the GdFeO_3_-type orthorhombic perovskites, as a prototype of the so-called ″locked-tilt″ perovskites.^1^ This compound represents the first finding of an orthorhombic perovskite characterized by the absence of changes in the octahedral tilt and a volume reduction with pressure controlled exclusively by an isotropic compression of the constituent polyhedral sites.

In recent years, it has become increasingly clear that a polar state and even ferroelectricity in centrosymmetric perovskite structures arise from an octahedral tilt mismatch between blocks of layered perovskites or at the interface of perovskite oxides and superlattices grown as thin films.^2^ Indeed, the strong coupling of oxygen rotations to the functional properties of a perovskite compound represents an opportunity to understand and produce new functional materials (e.g., multiferroics) that respond to external perturbation. Theoretically, this coupling can be controlled with a high degree of accuracy. From an experimental point of view, the mutual interaction between layers of octahedrally tilted perovskites subjected to an external perturbation could be tuned just as easily if one of the layers is a locked-tilt perovskite.

Perovskite oxides (general formula *AB*O_3_) have an ideal aristotype cubic structure (s.g. *Pm*3̅*m*) and, according to the so-called *B*-cell setting, can be described as *A* cations located in the center of dodecahedral sites defined by a three-dimensional (3D) framework of corner-sharing *B*O_6_ octahedra.^3^ When orthorhombic (s.g. *Pnma*), perovskites derive from the aristotype structure through a combination of octahedral tilts and distortions of the *B*O_6_ octahedra.^3−6^

GdFeO_3_-type perovskite minerals and synthetic analogues are widely studied by earth and materials scientists. Indeed, (Mg,Fe)SiO_3_ bridgmanite is the dominant phase of the Earth’s lower mantle.^7,8^ The first occurrence of a CaSiO_3_ perovskite that has retained its high-pressure orthorhombic structure at the Earth’s surface was reported in 2018.^9^ The mineral was trapped as an inclusion in a kimberlitic superdeep diamond, along with approximately 6% by volume of the orthorhombic CaTiO_3_ perovskite. The latter evidence highlights the presence of CaTiO_3_ at the mantle conditions and corroborates experimental studies on its stability.^10,11^

Excess physical properties, a consequence of structural phase transitions that distort the ideal aristotype structure, promote perovskites to reference compounds in several technological applications.^2,12−14^ The chemical composition, as well as changes in the octahedral tilting, are at the origin of these myriad physical properties (such as magnetic, electronic, electrical, and orbital properties) and are the basis for interpreting the variation of the perovskite state with temperature and pressure, as well as the thermodynamics associated with the possible occurrence of displacive phase transitions.^15^ In this context, many studies have been devoted to evaluating the behavior of GdFeO_3_-type perovskites and predicting their evolution at high pressure.^1,15−20^

The evolution of GdFeO_3_-type perovskites with pressure has been interpreted and formalized by a general rule as the combination of the relative compressibility of the constituent *A*O_12_ and *B*O_6_ polyhedra with the octahedral tilts.^15,18^ Namely, when *A* has a lower formal charge than *B* (i.e., 2+ and 4+, respectively; perovskites 2:4), the *A*O_12_ cubic site is more compressible than the *B*O_6_ octahedron and the volume reduction is associated with an increase in the octahedral tilt. Conversely, when *A* and *B* have the same formal charge (i.e., both cations are 3+; perovskites 3:3), *A*O_12_ is stiffer than *B*O_6_, and the volume reduction is partially compensated by a decrease in the octahedral tilt.^15^ Although true for most of the GdFeO_3_-type perovskites, the above description is incomplete and has recently been revised.^1,20,21^ In addition to defining dichotomous trends depending on the formal charge of *A* and *B* cations, the evolution of orthorhombic perovskites with pressure is influenced by whether transition metal ions (TMIs) are hosted at the octahedral site.^20,21^ The use of geochemical constraints (i.e., valence, ionic radius, diadochy rules) and the evaluation of the “normalized cell distortion factor with pressure, *d*_norm_(*P*)″, for several perovskite compounds,^1,19−22^ has allowed the identification of other possible locked-tilt perovskite formulations, viz., La(Mn_0.69_Ga_0.31_)O_3_, Ca(Ti_0.95_Ge_0.05_)O_3_, and (Sc_0.86_Y_0.14_)AlO_3_, respectively.

The purpose of this work is to investigate the high-pressure behavior of one of these candidates, namely, the Ca(Ti_0.95_Ge_0.05_)O_3_ perovskite. Besides a possible extension of the locked-tilt perovskite family, this investigation will provide a deeper understanding of the role of these compounds in view of their application as functional materials (e.g., multiferroics, layered perovskites).

## Experimental and *Ab Initio* Calculations

### Sample Description

A polycrystalline sample of Ge-doped CaTiO_3_ (CTG) with a perovskite structure was synthesized by a solid-state reaction using high-purity reagent-grade CaCO_3_, TiO_2_, and GeO_2_. The raw materials were mixed and homogenized by ball milling in acetone and then oven-dried at 100 °C. The dry powders were iteratively calcined in an unsealed alumina crucible at 1500 °C with a heating rate of 200 °C min^–1^ for 8 h until the preliminary X-ray diffraction analysis revealed a monophasic compound. Each calcination cycle was followed by natural cooling to room temperature.

### Synchrotron X-ray Powder Diffraction

#### Data Collection and Structure Refinement at Room Conditions (RT)

High-resolution synchrotron powder X-ray diffraction (HR-XRPD) data were collected at the ID22 beamline of the ESRF (Grenoble, France). The sample was placed in a spinning capillary rotating along the axis of the diffractometer at ∼2 Hz to improve crystallite statistics and was continuously scanned. HR-XRPD data were collected in parallel at 30.99 keV (λ = 0.40008 Å calibrated with the Si NIST standard SRM 640c) using nine scintillation detectors. After diffraction, the high-energy incident beam was passed through nine Si(111) analyzer crystals.^23^ The collected pattern was normalized, combined, and rebinned in a subsequent data reduction step to produce the equivalent step scan with a step size of 0.001°.^24^ X-ray diffraction patterns were recorded in the 0.5–38° 2θ range. Full-profile fitting analyses were performed using the fundamental-parameter (FP) Rietveld approach (TOPAS v5.0),^25^ starting from the structural model in reference.^26^ Known instrumental parameters (e.g., goniometer radius, slit sizes, geometrical parameters of the radiation source, etc.) were used to calculate the instrumental contribution to the peak profiles, and sample-related Lorentzian crystallite size and strain broadening information were extracted from the observed profiles. An instrumental zero error was fixed at the value determined using the Si NIST standard, and refinement included a sample displacement correction and a 12-term Chebyshev polynomial to model the background.

#### Data Collection and Pawley Fitting at High Pressure (HP)

The *in situ HP*-XRPD experiments were performed at the ID27 beamline of the ESRF (Grenoble, France). The hydrostatic pressure of up to 24 GPa was generated using a diamond anvil cell (DAC) with diamond culet surfaces of a 250 μm diameter. A small aliquot of the sample was loaded into a 65 μm diameter preindented gasket hole (i.e., a 40 μm thick Rh foil) using He as the pressure-transmitting medium.^27^ A small gold chip was placed near the sample as a pressure calibrant.^28^ The wavelength of the monochromatic incident X-ray beam was λ = 0.3738 Å, corresponding to the iodine K*-*edge. Debye–Scherrer rings were collected with a MAR165 CCD detector. The collected data were then reduced and radially integrated over the full rings by the DIOPTAS program,^29^ using the beam center, detector tilt, and sample-to-detector distance determined from a powder diffraction pattern of CeO_2_ as a standard to obtain the conventional intensity vs 2θ angle patterns.

It should be noted that the occurrence of highly spotted Debye–Scherrer rings hindered the extraction of well-resolved powder diffraction patterns (Figure S1 in the Supporting Information). This phenomenon can be attributed to a nonhomogeneous crystallite size distribution of the synthesized sample and technical limitations, in particular to the very narrow incident pencil beam used for the experiments. In fact, although collected with comparable beam energy (∼33 and ∼31 keV for XRPD experiments at the ID27 and ID22 beamlines, respectively), the data obtained from the RT experiment at ID22 benefited from technical features that cannot be implemented in HP experiments due to the physical limitations of membrane-driven DACs to generate high pressure. In particular, data collection on the ID22 beamline benefited from high sample illumination (∼1 mm^2^) and, most importantly, from the 9-channel Si(111) multianalyzer detector stage. Each Si(111) crystal has a very small angle acceptance (on the order of a few arcseconds), and the analyzer crystal strictly defines the 2θ diffraction angle without peak aberrations instead of deriving the angle from a pixel on a position-sensitive detector (as that used in HP experiments).^23^ Further technical details can be found on the beamline web pages.^30,31^ Therefore, due to limitations in both sample synthesis and experimental setup, only unit-cell parameters from HP diffraction experiments could be extracted by a whole powder pattern decomposition (WPPD) performed with a Pawley profile fit^32^ using TOPAS v. 5.0 software. Table S1 in the Supporting Information lists the unit-cell parameters (*a*, *b*, *c*, and *V*) of the CTG perovskite (s.g. *Pbnm*) up to 24 GPa.

### X-ray Absorption Spectroscopy

The powder sample was compressed into a pellet, and then Ge K-edge XAS spectra were collected at the XAFS beamline (ELETTRA, Trieste, Italy)^33^ in transmission mode, using a fixed exit Si(111) monochromator. Energy calibration was performed by simultaneously collecting a reference spectrum from a Ge^0^ pellet, placed in a second experimental chamber after the sample, with the position of the first inflection point taken at 11,103.0 eV. The data were collected at room temperature and in air using a variable step as a function of energy. Namely, a step of 5 eV in the first 200 eV of the spectrum, a step of 0.2 eV in the X-ray absorption near-edge structure (XANES) region, and a *k*-constant step of 0.03 Å^–1^ in the extended X-ray absorption fine structure (EXAFS) region. Multiple spectra were collected and merged in order to increase the signal-to-noise ratio and then normalized with respect to the high-energy side of the curve using Athena software.^34^ The contribution of the edge jump to the pre-edge was accounted for by subtracting a background function. The EXAFS signal was extracted with Athena and Fourier-transformed using a Hanning window in the *k*-range 3–12 Å^–1^, and quantitative analysis was performed using the Artemis software (Demeter 0.9.25 package).^34,35^

### Computational Strategy and Density Functional Theory (DFT) Calculations

**Figure 1:**
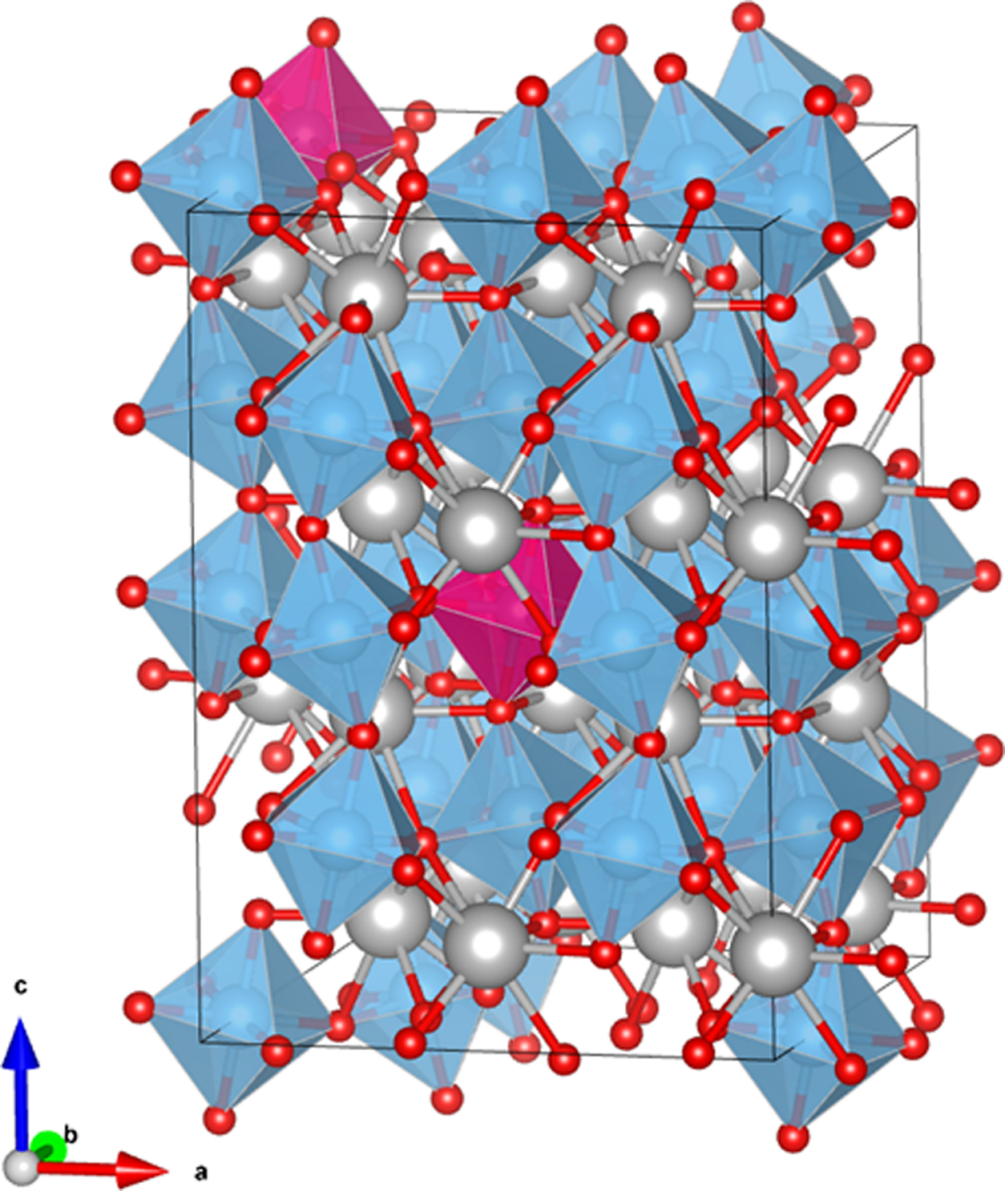
Supercell model structure of the Ca(Ti_0.94_Ge_0.06_)O_3_ perovskite, as derived from DFT calculations. Blue and pink octahedra represent Ti and Ge coordination polyhedra, respectively. Ca atoms are light gray, and O atoms are red.

In order to simulate the structure–energy properties of the CTG perovskite phase investigated in this work, a supercell model structure was created starting from the refined unit-cell parameters of the experimental structure (orthorhombic, *Pbnm*). A 2 × 2 × 2 supercell structure was first created for the CaTiO_3_ composition, and then atomic substitution of Ge for Ti was performed to simulate a composition close to that of the studied solid solution. Since the orthorhombic unit-cell of the CaTiO_3_ perovskite has *Z* = 4, the 2 × 2 × 2 supercell model structure contains 160 atoms, with 32 atomic positions occupied by Ti. Among all of the possible solid solution compositions that can be simulated by substituting Ge atoms for Ti in the 2 × 2 × 2 supercell, the composition closest to the experimental one was selected. In particular, a perovskite of the composition Ca(Ti_0.94_Ge_0.06_)O_3_ was selected in our structural model, with 1/16 of the atomic positions of Ti in the supercell occupied by Ge (Figure 1). The Ge occupancy in the octahedral sites of the simulated structure (i.e., 0.063 apfu) is thus close to that refined for the experimental structure, i.e., 0.055(1) apfu (see the “Results and Discussion” section). The Ti–Ge-substituted supercell structure of 160 atoms was fully relaxed in the *P*1 space group at *P* = 0 GPa and *T* = 0 K, and both atomic coordinates and cell parameters were optimized by variable-cell structure relaxation. Since a full configurational analysis of the perovskite phase with 6 atom % of Ge is far beyond the scope of the present work, the two Ge atoms were placed in the supercell in such a way as to minimize their near-neighbor interactions (i.e., to maximize their distances), in accordance with the experimental evidence from X-ray absorption spectroscopy, which makes clustering of Ge atoms in the structure unlikely (see the “Results and Discussion” section).

Density functional theory (DFT) calculations were performed with the Quantum ESPRESSO code^36,37^ using standard ultrasoft pseudopotentials from the GBRV library.^38^ A plane wave/density cutoff of 45/450 Ry and a 6 × 6 × 4 *k*-point grid were used, sufficient to converge the total energy by 1 mRy. The supercell structures of 160 atoms were fully relaxed at constant pressure, and both coordinates and cell parameters were optimized by variable-cell structure relaxations. All calculations were performed with the PBEsol exchange-correlation functional^39^ since GGA-based density functionals are known to provide equations of state parameters in very good agreement with experimental data obtained for dense oxide structures.^40,41^

Table S2 in the Supporting Information lists the pressure–volume–energy DFT values, along with unit-cell parameters (i.e., *a*, *b*, *c*, α, β, and γ) of the supercell model, while Tables S3–S14 list the atomic fractions (i.e., *x*, *y*, and *z*) of optimized structures as computed by the QE code in the pressure range from 0 to 25 GPa.

## Results and Discussion

### X-ray Absorption Spectroscopy

**Figure 2:**
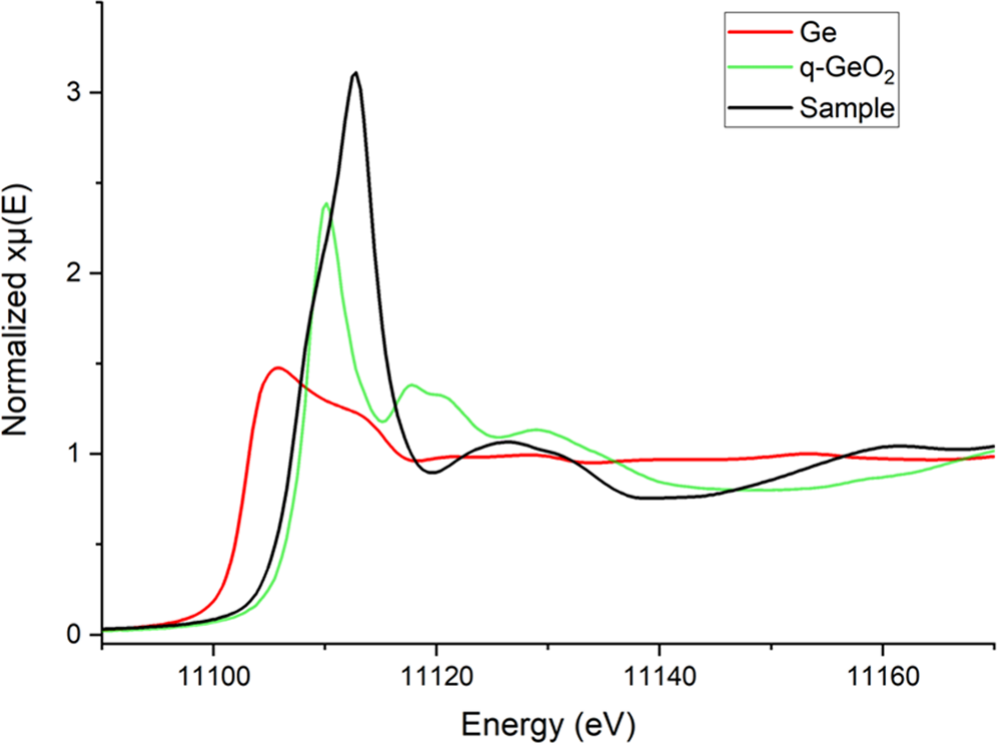
Normalized Ge *K*-edge XANES spectra for the CTG perovskite sample (black), Ge^0^ (red), and quartz-GeO_2_ (green) as reference compounds.

Figure 2 shows the normalized XANES spectra of the CTG and reference compounds.

**Figure 3:**
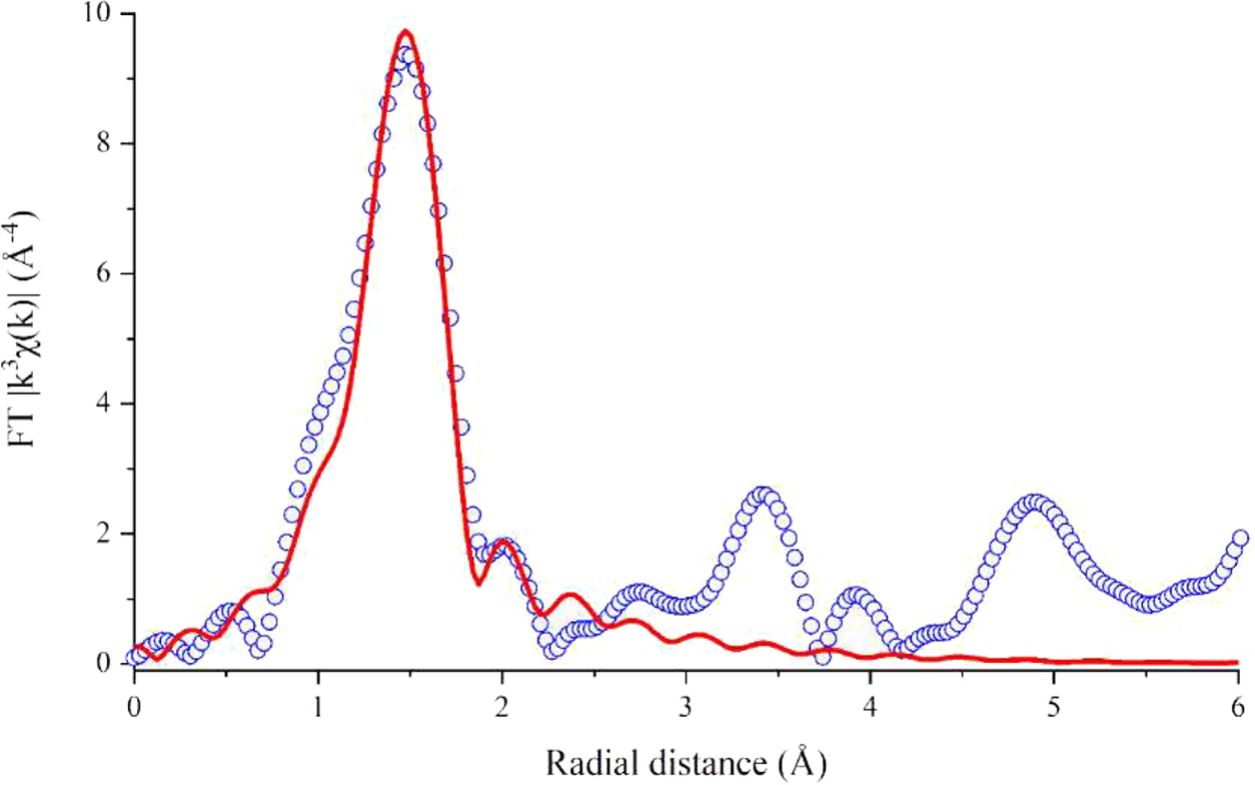
Fourier transform (FT) magnitude of the measured Ge K-edge EXAFS (circles) together with the result of the first shell modeling (red solid line) for the CTG perovskite.

The position of the absorption edge for Ge in CaTiO_3_ is comparable to that found in quartz-GeO_2_ (spectrum from the XAFS beamline database), suggesting that Ge in the sample under investigation is also tetravalent. However, differences in the features of the XANES region indicate a different coordination environment, suggesting a nontetrahedrally coordinated Ge as in q-GeO_2_.^42^ Indeed, the EXAFS refinement results for CTG (Figure 3 and Table 1) point to octahedrally coordinated Ge atoms.

**Table 1:** Crystallographic Data and Structure Parameters as Obtained from the EXAFS *R*-Space Fit^a^

shells	*N*	*R* (Å)	*S*_0_^2^	*R*-factor	σ^2^ (Å^2^)
Ge–O_2.1_	6	1.884(18)	0.7(1)	0.012	0.007(2)

a Shells calculated from the perovskite structure model in ref (26). *N*: coordination number; *R*: mean bond length; *S*_0_^2^: amplitude factor; and σ^2^: Debye–Waller thermal parameter.

The refined bond length of 1.88 Å is perfectly consistent with that expected for an octahedrally coordinated Ge^4+^ and very poorly agrees with that of a Ge^4+^ at a tetrahedral site (1.88 and 1.74 Å, respectively).^43^

### CTG Crystal Structure at Ambient Conditions

**Figure 4:**
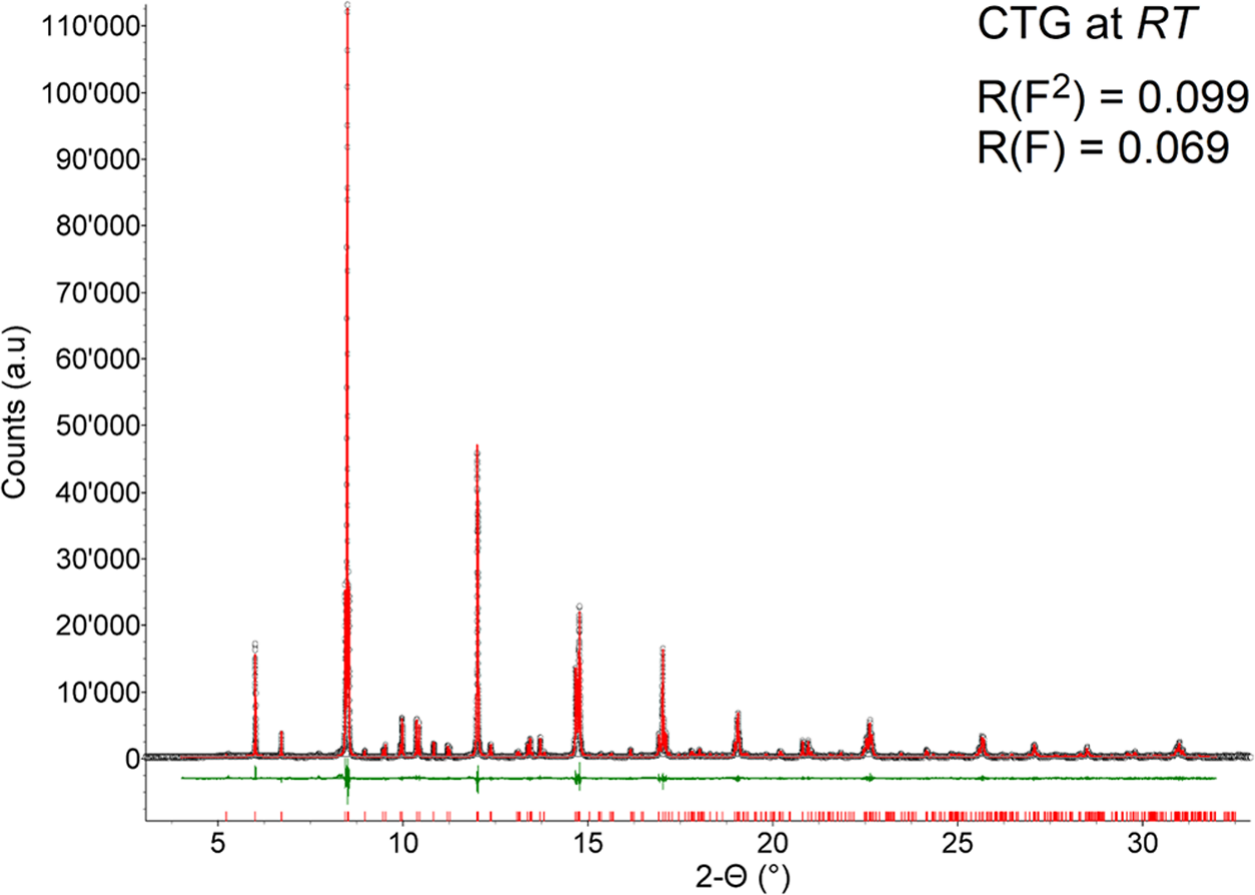
Rietveld refinement plot for the CTG perovskite as recorded under room conditions at the ESRF beamline ID22. The experimental data are indicated by circles (black), the calculated pattern is the solid line (red), and the lower curve (green) is the weighted difference between the observed and calculated patterns. Vertical ticks mark the position of the reflection for the perovskite in the *Pbnm* space group.

The Rietveld refinement plot of the HR-XRPD data collected at room conditions (Figure 4) shows that under our synthesis conditions, the perovskite compound is monophasic and crystallizes in the *Pbnm* space group as for the CaTiO_3_ isotype.^26^ The *B*O_6_ octahedra are tilted along the three directions of the cubic aristotype unit-cell, with a tilt system *a*^–^*a*^–^*c*^+^ in Glazer’s (10) notation.^5,6^

The refined unit-cell parameters (i.e., *a* = 5.38097(2) Å, *b* = 5.43780(2) Å, *c* = 7.64012(3) Å, and *V* = 223.555(1) Å^3^), as well as the bond distances and angles, are in perfect agreement with those of CaTiO_3_ perovskites as reported in the Inorganic Crystal Structure Database (comparison of selected data is reported in Table S15). It is worth noting that the refined atomic fraction of cations hosted at the octahedral site (i.e., Ti^4+^/Ge^4+^ = 0.945:0.055 apfu/apfu) is very close to the designed value in the batch composition. Although the cation substitution at the octahedral site is at the doping level, the presence of Ge^4+^, which has an ionic radius smaller than Ti^4+^ (i.e., 0.53 and 0.605 Å for octahedrally coordinated Ge and Ti, respectively),^44^ promotes *B*–O bond distances among the shortest for isochemically orthorhombic perovskites (Table S15), and consequently a smaller *B*O_6_ volume. This is in agreement with the bond distances from the EXAFS R-space fit (Table 1).

Comparing the structural data obtained from the experimental analyses with those obtained from the DFT calculations, it can be noted that although the structural optimization of the supercell model structure was performed in the *P*1 space group due to the Ti–Ge atomic substitutions, the final optimized structure at the athermal limit exhibits cell parameters similar to those of an orthorhombic cell, namely, the values of the cell angles close to 90° (i.e., α = 90.01°, β = 89.99°, and γ = 89.97°). A further check of the robustness of our structural model is obtained by comparing the total energy of the fully relaxed structure with that computed for a different configuration of Ge atoms in the supercell structure. Structure–energy calculations performed at any given pressure condition emphasize that the supercell model structure used in this work is always energetically favored over structural configurations with Ge atoms closer to each other in the supercell structure (see Table S2 in the Supporting Information).

### Elastic Properties and EoS of CTG

**Figure 5:**
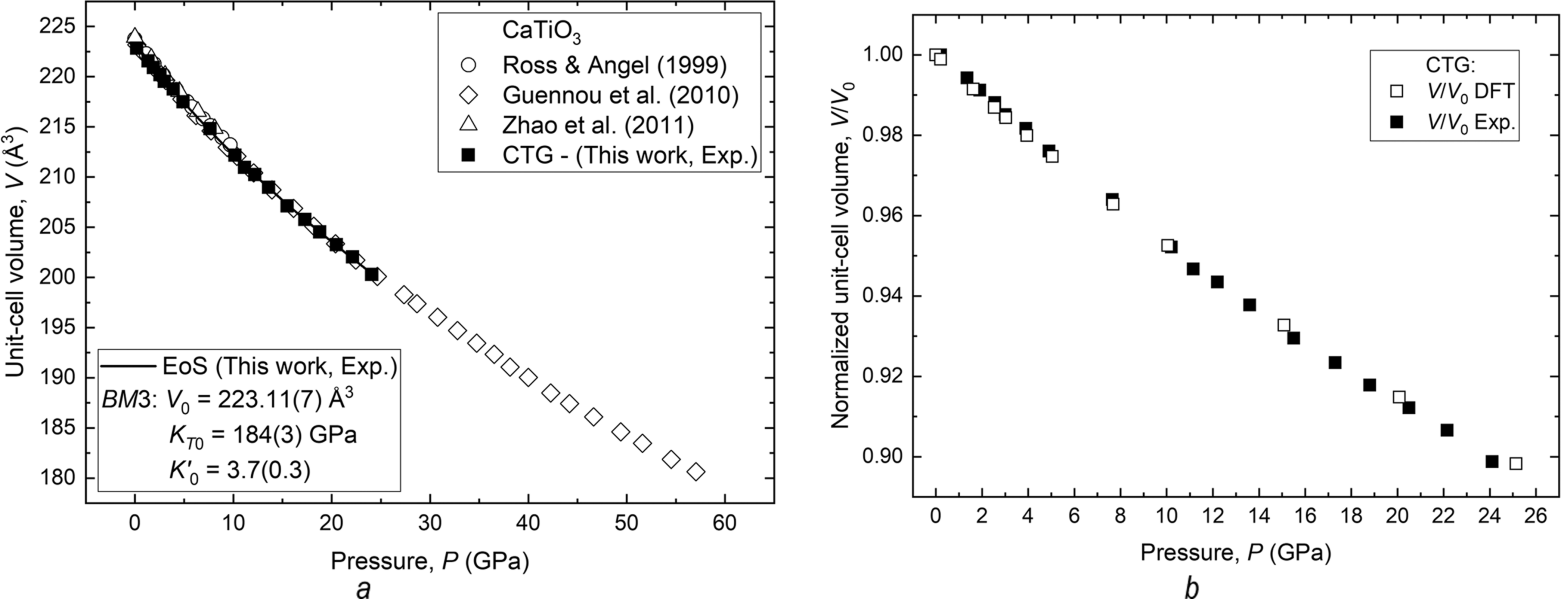
(a) Pressure dependence of the experimentally obtained unit-cell volume for CTG and other CaTiO_3_ perovskites from the literature (diamonds, circles, and triangles represent *P*–*V* experimental data from refs (11,45,46) respectively) and (b) comparison of the unit-cell volume variation with pressure (normalized to room pressure values) of data derived from XRD experiments and DFT calculations. The symbol sizes exceeded the estimated uncertainties. The solid line is the EoS fit to the HP data for the CTG sample.

The isothermal pressure dependence of the unit-cell volume for CTG (from experiments and DFT calculations) and other CaTiO_3_ orthorhombic perovskites from the literature is shown in Figure 5.

In analogy to the *P–V* data trends defined by the CaTiO_3_ compounds, the variation of the data for the CTG is continuous and smooth, and no evidence of phase transitions is observed in the pressure range under investigation.

Fitting the *P–V* data obtained for the CTG to a third-order Birch–Murnaghan equation of state (*BM*3-EoS) with EosFit7-GUI^47^ yielded the following EoS parameters: *V*_0_ = 223.11(7) Å^3^, a volumetric bulk modulus *K*_*T*0_ = 184(3) GPa, and a pressure derivative *K*′_0_ = 3.7(0.3). The unit-cell volume of the Ca(Ti_0.94_Ge_0.06_)O_3_ perovskite phase simulated by DFT calculations is slightly underestimated (∼2%) when compared to that refined for the experimental CTG phase [i.e., *V*_0_ = 219.645 Å^3^ vs 223.555(1) Å^3^]. Nevertheless, the *ab initio* static EoS parameters (i.e., *K*_*T*0_ = 187 GPa and *K*′_0_ = 4.2) are in very good agreement with the experimentally obtained values, also taking into account the fact that vibrational effects can lower the calculated values of the bulk modulus by several GPa even at ambient conditions, as shown by previous computational studies on oxide phases.^48^

The results of the data fitting with a *BM*3-EoS least-squares refinement for the CTG sample and other orthorhombic perovskites with the composition CaTiO_3_ are given in Table 2. A comparison of the data shows that CTG has volumetric compressibility close to that reported for CaTiO_3_ by Guennou and coauthors.^11^

**Table 2:** EoS Parameters Derived from a Data Fit to 3rd-Order Birch–Murnaghan EoS (*BM*3-EoS) for the Pressure Dependence of Unit-Cell Parameters for CTG and other Orthorhombic Perovskites with the Composition CaTiO_3_

	*K*_*T*0_ (GPa)	*K*_*a*0_ (GPa)	*K*_*b*0_ (GPa)	*K*_*c*0_ (GPa)			
	*K*′_0_	*K*′_*a*0_	*K*′_*b*0_	*K*′_*c*0_	*P*_max_ (GPa)	EoS	refs
CTG	183.8(2.8)	185.3(2.6)	182.6(3.5)	182.0(4.3)	24.1	*BM*3	this work; Exp.
	3.7(0.3)	3.5(0.2)	3.8(0.3)	3.9(0.4)			
CTG	187.0(0.1)	189.7(1.7)	185.4(2.2)	184.2(1.0)	25.1	*BM*3	this work; DFT
	4.2(0.1)	2.9(0.1)	5.5(0.2)	4.7(0.1)			
CaTiO_3_	170.9(1.4)	168.7(2.1)	168.3(1.9)	175.3(1.5)	9.7	*BM*3	(45)
	6.6(3)	5.7(5)	7.0(4)	6.6(3)			
CaTiO_3_	181.0(6)	174.5(2.3)	185.8(1.0)	182.6(1.1)	40.0	*BM*2	(11)
CaTiO_3_^a^	170.1(3.1)	170.5(3.1)	166.6(3.0)	173.0(6.5)	8.1	*BM*3	(46)
	6.8(9)	5.2(9)	8.0(9)	7.8(1.9)			

a Note: Data fitting was not reported by the authors but refined using EosFit7-GUI^47^ for comparison.

**Figure 6:**
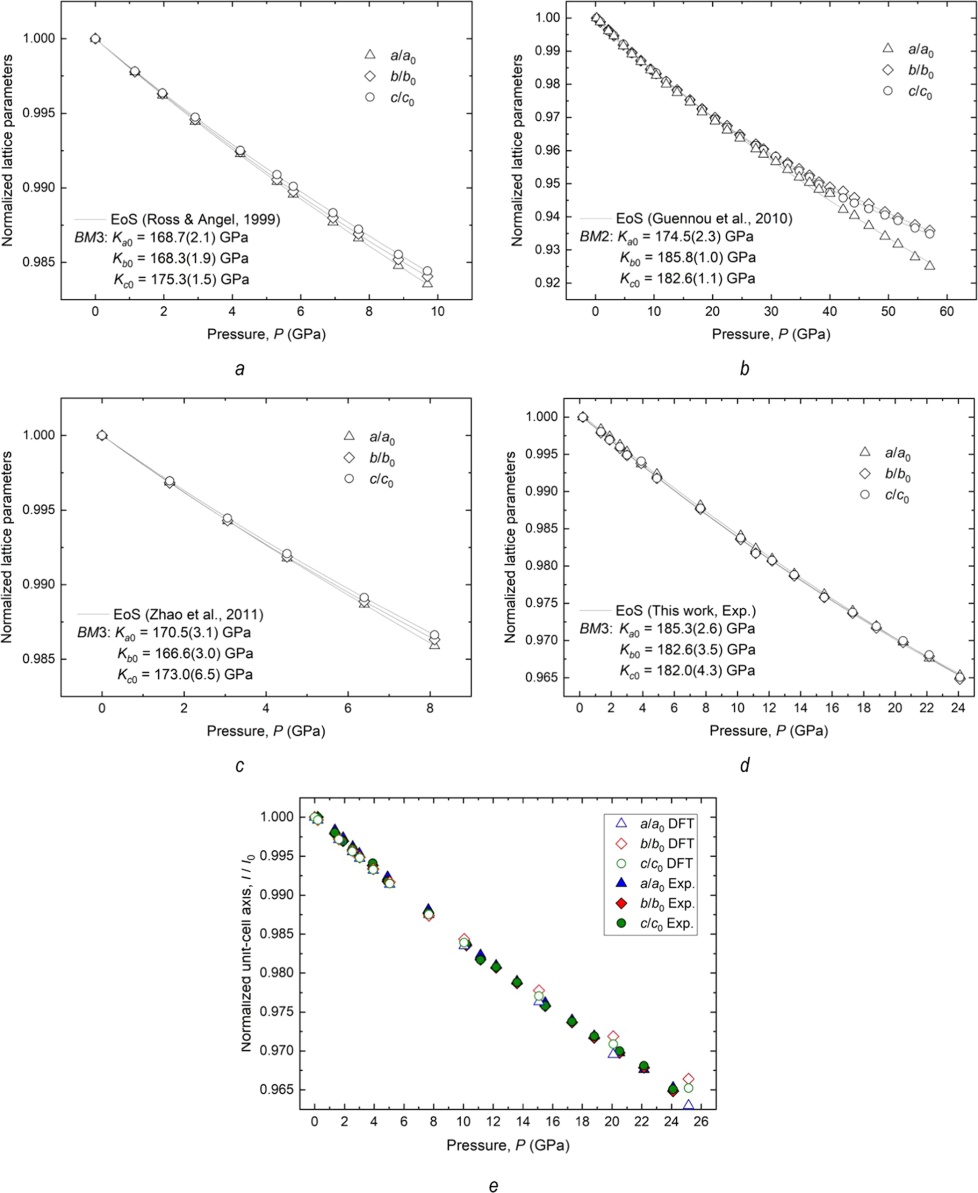
Pressure dependence of normalized unit-cell axes for CaTiO_3_ perovskites from the literature (a–c),^11,45,46^ and that for CTG (d) as derived from X-ray diffraction experiments. (e) Variation of unit-cell parameters with pressure (normalized to room pressure values) of data derived from XRD experiments and DFT calculations. The symbol sizes exceed the estimated measurement uncertainties. The solid line is the EoS fit to the HP data. The data fit for CaTiO_3_ in panel (d) was not reported by the authors but was refined in this work using the EosFit7-GUI^47^ for comparison.

In contrast to the other high-pressure studies on CaTiO_3_ orthorhombic perovskites, where the compression regime produced an anisotropic response along the three axial directions (Figure 6a–c), resulting in different axial bulk moduli along the *a*, *b*, and *c* axes (Table 2); compression along the three axial directions for CTG defines trends that overlap within the data uncertainties (Figure 6d). This implies that the substitution of Ge for Ti at the octahedral site results in an orthorhombic perovskite structure that compresses isotropically. Indeed, data fitting of the unit-cell axes to *BM*3-EoS yielded identical axial moduli within the standard deviation (Table 2). The normalized axial compressibilities (i.e., *a*/*a*_0_, *b*/*b*_0_, and *c*/*c*_0_) computed by DFT-GGA calculations confirm the trends observed by experiments up to about 10 GPa (Figure 6e), after which slight deviations may occur, possibly due to enhanced Ge atomic interactions in the supercell model at higher pressures. DFT calculations thus confirm that the substitution of Ge for Ti in the octahedral sites of the perovskite structure makes its compressibility nearly isotropic.

### Normalized Cell Distortion Factor with Pressure of CaB^4+^O_3_ (B^4+^: Ti and Ge) Compounds

A very useful and visually effective way to assess the pressure dependence for a perovskite crystal lattice is through the ″normalized cell distortion factor with pressure,″ *d*_norm_(*P*).^1,19,20^ The *d*_norm_(*P*) is defined as the isothermal rate of change of the cell distortion factor (*d*) with pressure, as defined by Sasaki et al.,^49^ normalized to the value calculated at ambient conditions (*d*_0_), such that
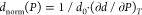
1

**Figure 7:**
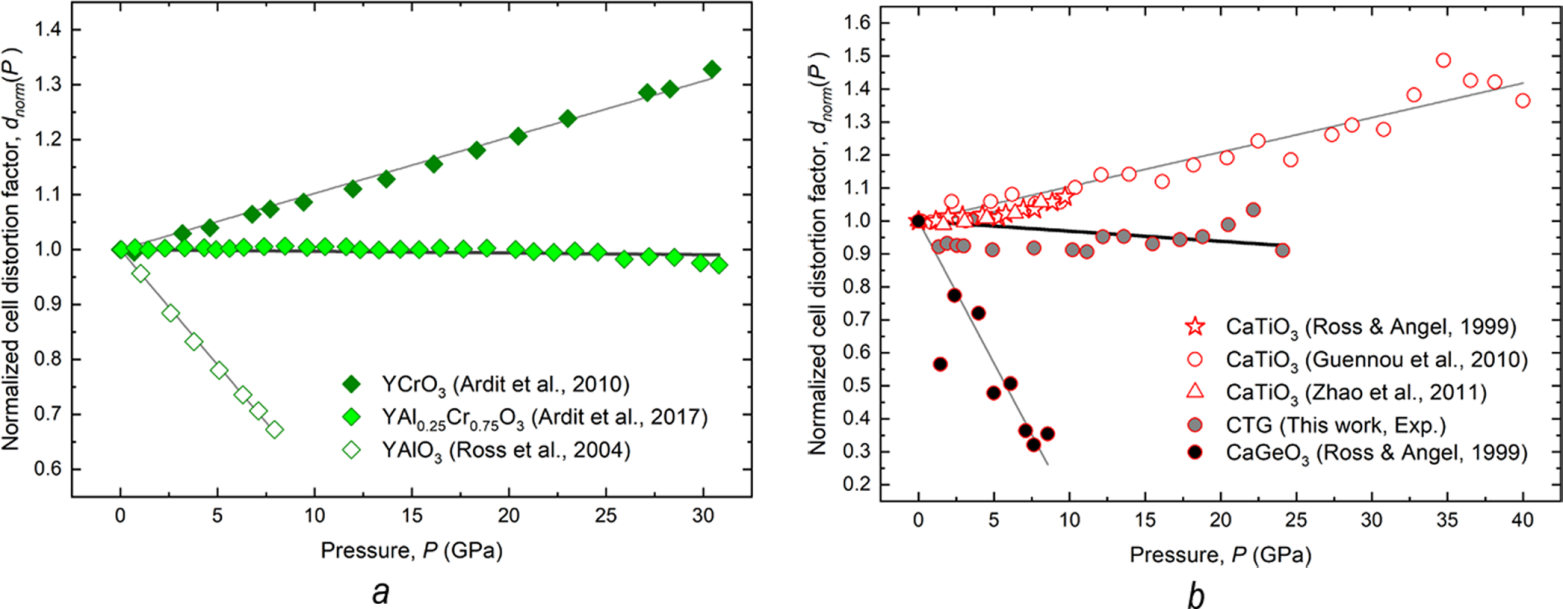
Normalized cell distortion factor with pressure, *d*_norm_(*P*), for YM^3+^O_3_ (*a*) and CaB^4+^O_3_ (*b*) orthorhombic perovskites, as derived from X-ray diffraction experiments. The solid lines are linear fits to the data. In panel (a), data for the isotypic series YM^3+^O_3_ (M = Cr and Al) are from.^1,50,51^ In panel (b), data for the isotypic series CaB^4+^O_3_ (B = Ti and Ge) are from this work and.^11,45,46^

In analogy to what was previously observed for 3:3 orthorhombic perovskites belonging to the YM^3+^O_3_ (M^3+^: Al and Cr) series (graphical summary in Figure 7a; for a detailed description, see ref (1)), 2:4 perovskites belonging to the CaB^4+^O_3_ (B^4+^: Ti and Ge) series exhibit all of the possible lattice evolutions with pressure for orthorhombic perovskites (Figure 7b).

Specifically, three different cases can be identified based on the *d*_norm_(*P*) evolution with pressure:

(1) As predicted by the general rule for the HP evolution of GdFeO_3_-type perovskites,^15,18^ CaTiO_3_ perovskites are characterized by an increase in lattice distortion with pressure (empty symbols in Figure 7b). In these compounds, volume reduction due to polyhedral site compressibility, with *K*(*A*O_12_) < *K*(*B*O_6_), is enhanced by an increase in the octahedral tilting.^46^*Ab initio* DFT first-principles calculations showed that second-order phase transitions to higher symmetries are impossible for CaTiO_3_ under high pressure at RT and further predicted a phase transition to a postperovskite crystal structure (s.g. *Cmcm*) at sufficiently high temperature and pressure.^52^
(2) In contrast to CaTiO_3_, CaGeO_3_ seems to violate the general rule of pressure dependence for 2:4 orthorhombic perovskites. Indeed, the lattice distortion with pressure for the latter compound decreases with pressure (*d*_norm_(*P*) → 0), indicating that the volume reduction due to differential polyhedral compressibility, *i.e., K*(*A*O_12_) > *K*(*B*O_6_), as predicted for 3:3 orthorhombic perovskites,^15,18^ would be partially compensated by a decrease in the octahedral tilting. Therefore, a symmetry reduction should occur at pressures higher than those investigated by previous works (where symmetry changes were not detected).^44^ On the other hand, the experimental evidence reported by Ross and Angel^44^ led to data trends in Figure 7b that would contrast with more recent experimental (HP XRD experiments) and theoretical (DFT first-principles calculations) investigations.^53^ The results reported in the latter study indicate that the CaGeO_3_ perovskite becomes more distorted with increasing pressure. In addition, a phase transition to a postperovskite structure is predicted to occur in the 36–44 GPa *P*-range. However, the HP experimental data for CaGeO_3_ by Wu et al.^53^ are highly dispersed and show a highly scattered *d*_norm_(*P*) evolution with pressure when compared to other orthorhombic perovskites along the CaB^4+^O_3_ (*B*^4+^: Ti and Ge) series (Figure S2). Indeed, the authors reported anomalous trends for lattice parameters at high pressure and that the accuracy of the results was hampered by a diffraction peak broadening attributed to a deviatoric stress increase with pressure.^53^
(3) CTG (as YAl_0.25_Cr_0.75_O_3_ for 3:3 perovskites along the YAl_1–*x*_Cr_*x*_O_3_ series) is located in the middle. Whatever the compression regime suffered, this perovskite maintains its lattice distortion unchanged, equal to its initial value at ambient conditions.

### High-Pressure Structure Evolution of CTG by DFT-GGA Calculations

Since the interplay between the relative compressibility of the *A*O_12_ and *B*O_6_ polyhedral sites and the *B*O_6_ octahedral tilt plays a key role in determining whether the perovskite structure becomes more distorted with increasing pressure, in order to qualify CTG as the first 2:4 locked-tilt perovskite, the structural evolution (i.e., the change in bond lengths, polyhedral volumes, and octahedral tilt) with pressure must be carefully studied. Due to the limitations of high-pressure experiments (see the Experimental section), the structural evolution of the CTG perovskite is studied using data derived from DFT-GGA calculations on the Ca(Ti_0.94_Ge_0.06_)O_3_ structural model. It should be emphasized that theoretical predictions of high-pressure structural evolution are based entirely on static energy calculations and that neither configurational entropy contributions nor thermal effects have been considered in this analysis. In fact, ignoring these effects does not significantly alter the validity of the outcomes obtained by static DFT calculations due to the low-temperature conditions considered in this work (i.e., zero or ambient temperature) and the high degree of dilution of the CTG solid solution (i.e., 6 mol % of CaGeO_3_). For example, considering a random mixing of Ge atoms in the octahedral sites of the structure, the estimated value for the configurational entropy (*S*_conf_) of the Ca(Ti_0.94_Ge_0.06_)O_3_ phase would be equal to^54^*S*_conf_ = −*R*·∑_*i*_ [*X*_*i*_·ln(*X*_*i*_)] = [0.94·ln(0.94) + 0.06·ln(0.06)] = 1.887 J/mol·K ≈ 0.002 kJ/mol·K, where *X*_*i*_ is the molar fraction of the CaTiO_3_ or CaGeO_3_ component in the solid solution and *R* is the gas constant (*R* = 8.31446 J/mol·K). The estimated entropy results in a contribution to the Gibbs free energy of mixing of the phase of about −0.56 kJ/mol, which is negligible as compared to the total energy computed for the CTG phase at the static level (i.e., *E*_0_ = −390,263.26 kJ/mol) or, alternatively, to its molar Gibbs free energy at the standard state (i.e., ∼0.03% of *G*_0_). As for thermal effects, previous DFT investigations, even on structurally complex compounds,^55,56^ support the evidence that they are not relevant enough to significantly change compressibility trends at room temperature.

**Figure 8:**
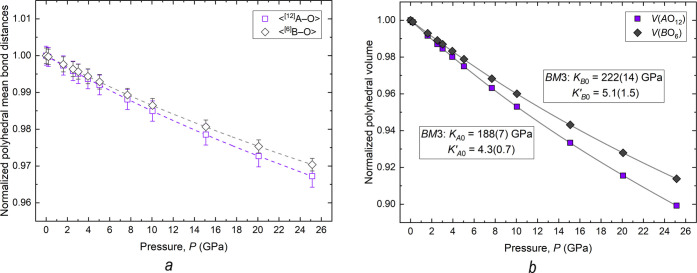
Pressure dependence of normalized polyhedral mean bond distances (a) and normalized polyhedral volumes (b) derived from DFT-GGA calculations. In panel (b), the symbol sizes exceed the estimated uncertainties, and the solid lines are the EoS fit to the HP data.

Figure 8 shows the pressure dependence of the ⟨^[12]^*A*–O⟩ and ⟨*B*–O⟩ mean bond distances (a) and for the polyhedral volumes *V*_A_ and *V*_B_ (b).

Although the compressibility of the bond distances of polyhedron *A* is comparable to that of polyhedron *B* within the estimated standard deviation (Figure 8a), it is evident from Figure 8b that the compressibility of the *B*O_6_ octahedra is greater than that of the *A*O_12_ polyhedra. The parameters derived from *P*–*V* data by fitting a *BM*3-EoS, i.e., the polyhedral bulk modulus (*K*_*P*0_) and its pressure derivative (*K*′_*P*0_), are 188(7) and 4.3(0.7) for the CaO_12_ cubic sites and 222(14) and 5.1(1.5) for the (Ti_0.94_Ge_0.06_)O_6_ octahedra, respectively.

**Figure 9:**
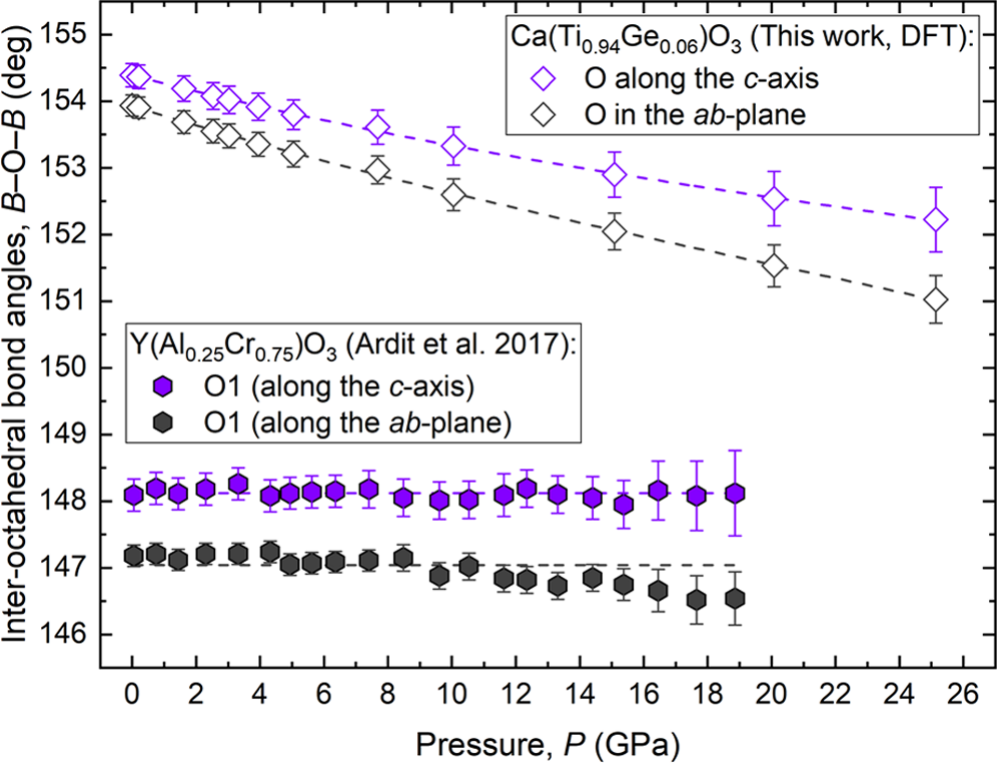
Pressure dependence of the interoctahedral bond angles for the Ca(Ti_0.94_Ge_0.06_)O_3_ and Y(Al_0.25_Cr_0.75_)O_3_ perovskite structures as derived from DFT-GGA calculations and X-ray diffraction experiments, respectively.

The reason for the anisotropy in the polyhedral compressibility can be attributed to the progressive increase in the tilt of the octahedra with increasing pressure. As shown in Figure 9, unlike the perovskite YAl_0.25_Cr_0.75_O_3_, which is characterized by the absence of variation in the *B*–O–*B* bond angles,^1^ both the interoctahedral angles in the *ab*-plane and those along the *c*-axis for the Ca(Ti_0.94_Ge_0.06_)O_3_ structure deviate more and more from the ideal value of 180° with increasing pressure.

## Concluding Remarks

The multimethodological approach used in this work, where experimental results from synchrotron radiation analyses were complemented by data from DFT-GGA calculations, allowed us to characterize the structural evolution at high-pressure of a Ge-doped CaTiO_3_ compound (CTG) with a perovskite structure.

The partial substitution of Ti by Ge at the octahedral site modifies the lattice response to compression of the perovskite CaTiO_3_, rendering it isotropic (the same compressibility is observed along the three unit-cell axes *a*, *b*, and *c*) and confirming that the use of geochemical constraints (i.e., valence, ionic radius, diadochy rules, tolerance factor) can help in predicting perovskite structures with a normalized cell distortion factor with pressure, *d*_norm_(*P*), close to 1. However, an increasing tilt of the octahedra, causing anisotropy in the polyhedral compressibility, is predicted by the evolution of the structural parameters derived from the DFT-GGA calculations, excluding the CTG sample as a possible member of the locked-tilt perovskite family, at least in the pressure range of validity of the supercell model used for CTG in this work.

The nearly isotropic compression behavior of CTG (i.e., *K*_*a*0_ = *K*_*b*0_ = *K*_*c*0_), confirmed by both experimental measurements and DFT calculations, raised the question of whether it contradicts the evidence suggesting that CTG is not a 2:4 locked-tilt perovskite. However, based on the combined findings from experimental and computational approaches, the former observation did not rule out the latter. The anisotropy in polyhedral compressibility, as revealed by *ab initio* DFT calculations, played a crucial role in understanding this aspect.

Indeed, the CTG compound can be considered the first example of a new type of GdFeO_3_-type perovskites where lattice compression isotropy (*d*_norm_(*P*) = 1) is associated with a modest change in octahedral tilting upon pressure (not locked-tilt). This type of compound complements the two existing ones: (1) GdFeO_3_-type perovskites with lattice compression anisotropy (*d*_norm_(*P*) ≠ 1) and change in the octahedral tilting on pressure (not locked-tilt) and (2) GdFeO_3_-type perovskites with lattice compression isotropy (*d*_norm_(*P*) = 1) and absence of variation in octahedral tilting (locked-tilt).

The synergy between synchrotron experiments and state-of-the-art DFT calculations was highlighted as the main way to establish a “protocol design” capable of effectively investigating complex problems in high-pressure research. This included understanding the physical behavior of locked-tilt perovskite systems under nonambient conditions. Importantly, the characterization strategy developed in this study can be adapted to explore other potential locked-tilt perovskite candidates.

## Supporting Information Available

Experimental section, data analysis, and additional crystallographic data (unit-cell axis values at high pressure from both synchrotron X-ray diffraction and DFT calculations and atomic coordinates obtained from the DFT calculation) (PDF)
