# Supplementary material for: Structure Evolution of Ge-Doped CaTiO3 (CTG) at High Pressure: Search for the First 2:4 Locked-Tilt Perovskite by Synchrotron X-ray Diffraction and DFT Calculations
Source: Inorg Chem. 2023 Oct 5;62(41):16943–53. doi: 10.1021/acs.inorgchem.3c02645 (PMC10583197; doi:10.1021/acs.inorgchem.3c02645)
Supplement: Supplementary file 1 — ic3c02645_si_001.pdf [file ic3c02645_si_001.pdf]

Structure evolution of Ge-doped $CaTiO_3$ (CTG) at high pressure: Search for the first 2:4 locked-tilt perovskite by synchrotron X-ray diffraction and DFT calculations

Matteo Ardit,[1] Sonia Conte,[2] Donato Belmonte,[3] Francesca Menescardi,[3] Simone Pollastri,[4] Giuseppe Cruciani,[1] and Michele Dondi[2]

[1]Department of Physics and Earth Sciences, University of Ferrara, via Saragat 1, I-44122 Ferrara, Italy

[2]CNR-ISSMC, Institute of Science, Technology and Sustainability for the Development of Ceramic Materials, via Granarolo 64, I-48018 Faenza, Italy

[3]Department of Earth Sciences, Environment and Life (DISTAV), University of Genova, corso Europa 26, I-16132 Genova, Italy

[4]ELETTRA - Sincrotrone Trieste, ss 14, km 163.5, I-34149 Basovizza, Italy

Authors: Matteo Ardit; orcid.org/0000-0001-8998-3063; Email: rdtmtt@unife.it

Sonia Conte; orcid.org/0000-0002-4746-4115; Email: sonia.conte@issmc.cnr.it

Donato Belmonte; orcid.org/0000-0002-6560-0248; Email: donato.belmonte@unige.it

Francesca Menescardi; orcid.org/0000-0002-5916-8727; Email: francesca.menescardi@edu.unige.it

Simone Pollastri; orcid.org/0000-0002-4730-7208; Email: simone.pollastri@elettra.eu

Giuseppe Cruciani; orcid.org/0000-0002-4269-697X; Email: cru@unife.it

Michele Dondi; orcid.org/0000-0001-6606-1146; Email: michele.dondi@issmc.cnr.it

Supplementary Material

Figures

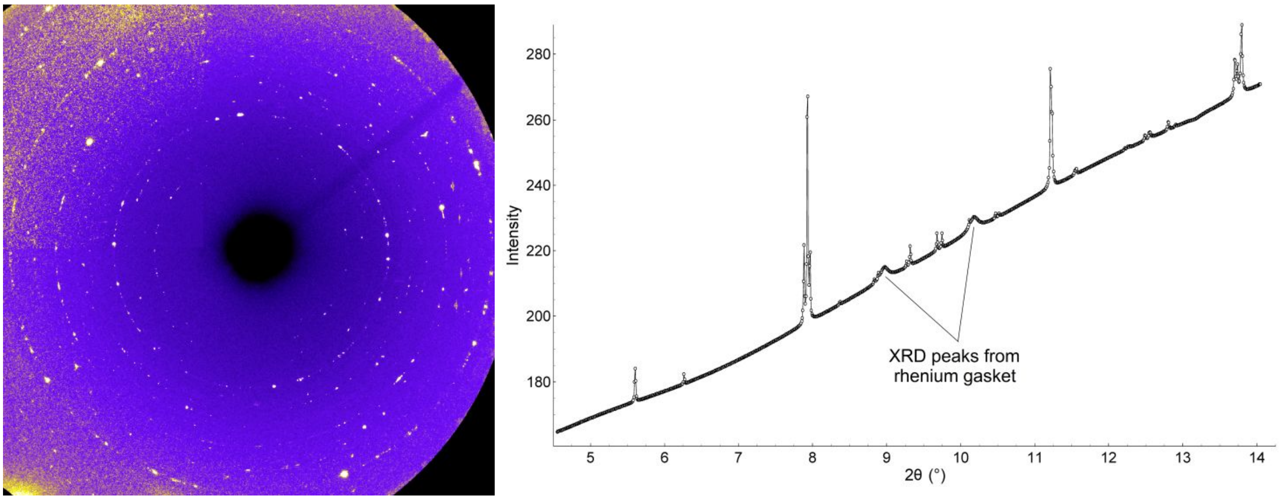

a *b*

Figure S1. Debye-Scherrer rings collected with the MAR165 CCD detector at the ID27 beamline (ESRF) (*a*), and data reduction by the DIOPTAS program (*b*) of the CTG sample at 0.20 GPa.

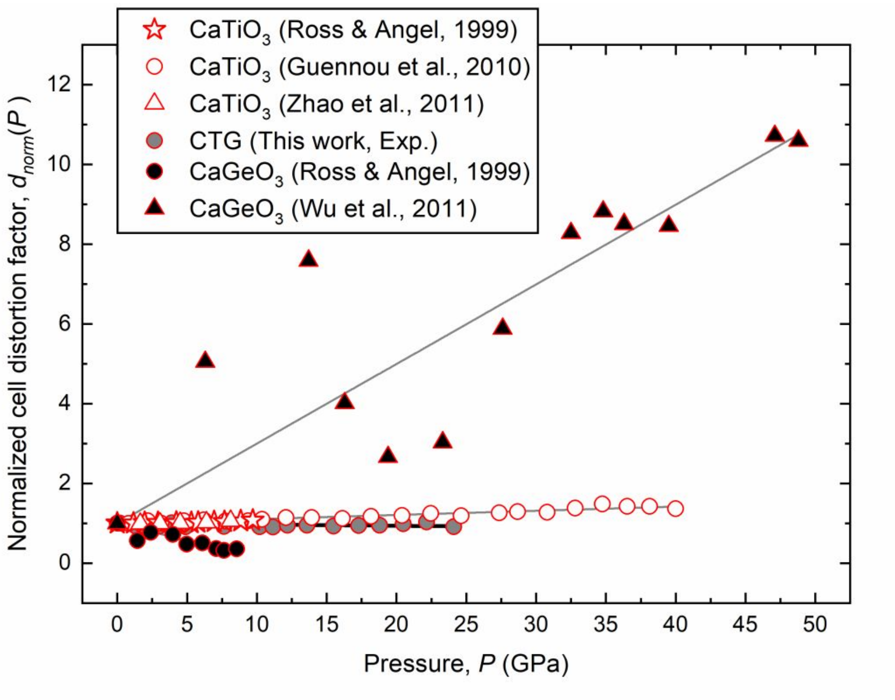

Figure S2. Normalized cell distortion factor with pressure, $d_{norm}(P)$, as a function of pressure for $CaB^{2+}O_3$ orthorhombic perovskites as derived from X-ray diffraction experiments. Solid lines are linear fits to the data. Data for the isotypic series $CaB^{2+}O_3$ (B = Ti and Ge) are from [1-4].

Figure S2 references

(1) Guennou, M.; Bouvier, P.; Krikler, B.; Kreisel, J.; Haumont, R.; Garbarino, G. High-pressure investigation of $CaTiO_3$ up to 60 GPa using x-ray diffraction and Raman spectroscopy. *Phys. Rev. B* **2010**, *82*(13), 134101.

(2) Ross, N. L.; Angel, R. J. Compression of $CaTiO_3$ and $CaGeO_3$ perovskites. *Am. Mineral.* **1999**, *84*(3), 277-281.

(3) Wu, X.; Qin, S.; Gu, T. T.; Yang, J.; Manthilake, G. Structural and elastic properties of $CaGeO_3$ perovskite at high pressures. *Phys. Earth Planet. In.* **2011**, *189*(3-4), 151-156.

(4) Zhao, J.; Ross, N. L.; Wang, D.; Angel, R. J. High-pressure crystal structure of elastically isotropic $CaTiO_3$ perovskite under hydrostatic and non-hydrostatic conditions. *J. Phys.-Condens. Mat.* **2011**, *23*(45), 455401.

Tables

Table S1. Unit-cell parameters (*a*, *b*, *c*, and *V*) of the CTG perovskite (s.g. *Pbnm*) up to 24 GPa derived from *HP* diffraction experiments and extracted by a whole powder pattern decomposition (WPPD) through a Pawley profile fit using TOPAS v. 5.0 software.

P (GPa)	*a* (Å)	*b* (Å)	*c* (Å)	*V* (Å^3)
0.20(5)	5.3758(7)	5.4330(6)	7.6293(6)	222.83(4)
1.35(8)	5.3670(4)	5.4216(4)	7.6143(4)	221.56(3)
1.90(5)	5.3617(4)	5.4164(4)	7.6059(4)	220.88(3)
2.55(8)	5.3556(5)	5.4103(5)	7.5988(5)	220.18(3)
3.00(5)	5.3507(5)	5.4051(5)	7.5905(5)	219.52(3)
3.90(10)	5.3419(6)	5.3994(5)	7.5842(5)	218.75(4)
4.90(5)	5.3346(7)	5.3883(7)	7.5665(7)	217.49(5)
7.65(8)	5.3120(7)	5.3659(7)	7.5360(7)	214.80(4)
10.20(5)	5.2901(8)	5.3437(8)	7.5055(8)	212.17(5)
11.15(7)	5.2808(7)	5.3337(7)	7.4896(7)	210.95(4)
12.20(10)	5.2735(7)	5.3281(7)	7.4825(7)	210.24(4)
13.60(10)	5.2626(7)	5.3172(6)	7.4677(7)	208.96(4)
15.50(10)	5.2479(6)	5.3014(6)	7.4446(6)	207.12(4)
17.30(10)	5.2359(7)	5.2899(6)	7.4293(7)	205.77(4)
18.80(10)	5.2249(8)	5.2792(7)	7.4149(8)	204.52(5)
20.50(10)	5.2133(7)	5.2687(6)	7.4003(6)	203.26(4)
22.15(13)	5.2017(6)	5.2584(5)	7.3859(6)	202.02(3)
24.10(5)	5.1893(5)	5.2419(5)	7.3628(6)	200.28(3)

Table S2. Pressure-volume-energy values and unit-cell parameters of the supercell model for the $Ca(Ti_{0.94}Ge_{0.06})O_3$ perovskite as obtained from DFT calculations up to 25 GPa.

P (GPa)	*V* (Å^3)	*E* (Ry)	*a* (Å)	*b* (Å)	*c* (Å)	*α* (°)	*β* (°)	*γ* (°)
0.004	1757.16	-9513.18	10.6652	10.8597	15.1714	89.974	89.990	90.011
0.206	1755.25	-9513.18	10.6613	10.8558	15.1659	89.974	89.990	90.011
1.623	1742.20	-9513.17	10.6346	10.8291	15.1282	89.976	89.990	90.011
2.530	1734.12	-9513.16	10.6179	10.8126	15.1047	89.978	89.990	90.011
3.032	1729.73	-9513.16	10.6088	10.8036	15.0919	89.978	89.990	90.011
3.942	1721.93	-9513.14	10.5927	10.7874	15.0691	89.979	89.990	90.011
5.038	1712.77	-9513.12	10.5734	10.7689	15.0422	89.981	89.990	90.011
7.679	1691.87	-9513.06	10.5319	10.7225	14.9819	89.979	89.987	90.014
10.055	1673.84	-9512.99	10.4895	10.6900	14.9274	89.989	89.990	90.011
15.079	1638.95	-9512.78	10.4128	10.6183	14.8232	89.996	89.990	90.012
20.081	1607.49	-9512.53	10.3404	10.5542	14.7295	90.002	89.989	90.013
25.141	1578.41	-9512.22	10.2706	10.4948	14.6437	90.007	89.989	90.015

Table SI3. Atomic coordinates obtained from the DFT calculation for the sample CTG at P = 0.004 GPa.

Atom label	Atom type	*x*	*y*	*z*	Atom label	Atom type	*x*	*y*	*z*	Atom label	Atom type	*x*	*y*	*z*
Ca1	Ca	-0.00489	0.02145	0.12512	Ti61	Ti	0.25000	0.00000	-0.25000	O121	O	-0.10406	-0.10424	0.22989
Ca2	Ca	-0.00443	0.02134	-0.37492	Ti62	Ti	0.25000	0.00000	0.25000	O122	O	-0.10399	-0.10475	-0.26956
Ca3	Ca	-0.00469	-0.47713	0.12428	Ti63	Ti	0.25000	-0.50000	-0.25000	O123	O	-0.10396	0.39576	0.22883
Ca4	Ca	-0.00415	-0.47805	-0.37536	Ti64	Ti	0.25000	-0.50000	0.25000	O124	O	-0.10408	0.39571	-0.27113
Ca5	Ca	0.49585	0.02203	0.12451	O65	O	0.04030	0.23965	0.12496	O125	O	0.39601	-0.10424	0.22881
Ca6	Ca	0.49538	0.02289	-0.37564	O66	O	0.03993	0.23990	-0.37501	O126	O	0.39611	-0.10419	-0.27104
Ca7	Ca	0.49552	-0.47862	0.12509	O67	O	0.03849	-0.25881	0.12258	O127	O	0.39600	0.39522	0.23038
Ca8	Ca	0.49515	-0.47838	-0.37481	O68	O	0.03849	-0.25946	-0.37458	O128	O	0.39602	0.39586	-0.27002
Ca9	Ca	0.00462	-0.02289	-0.12436	O69	O	-0.46145	0.24043	0.12538	O129	O	0.14292	-0.14730	-0.02023
Ca10	Ca	0.00415	-0.02202	0.37549	O70	O	-0.46158	0.24115	-0.37739	O130	O	0.14573	-0.14467	0.47978
Ca11	Ca	0.00485	0.47839	-0.12519	O71	O	-0.46006	-0.25999	0.12498	O131	O	0.14465	0.35599	-0.02125
Ca12	Ca	0.00448	0.47863	0.37491	O72	O	-0.45976	-0.26021	-0.37498	O132	O	0.14531	0.35496	0.47895
Ca13	Ca	-0.49557	-0.02133	-0.12508	O73	O	-0.03842	-0.24114	-0.12261	O133	O	-0.35475	-0.14495	-0.02117
Ca14	Ca	-0.49511	-0.02144	0.37488	O74	O	-0.03855	-0.24042	0.37462	O134	O	-0.35524	-0.14403	0.47884
Ca15	Ca	-0.49585	0.47805	-0.12464	O75	O	-0.04024	0.26022	-0.12502	O135	O	-0.35438	0.35540	-0.02037
Ca16	Ca	-0.49531	0.47714	0.37572	O76	O	-0.03994	0.26000	0.37502	O136	O	-0.35711	0.35273	0.47975
Ca17	Ca	0.24556	0.22775	-0.12469	O77	O	0.46006	-0.23990	-0.12499	O137	O	-0.14594	0.14563	0.22890
Ca18	Ca	0.24537	0.22812	0.37502	O78	O	0.45970	-0.23965	0.37504	O138	O	-0.14573	0.14541	-0.27108
Ca19	Ca	0.24491	-0.27127	-0.12490	O79	O	0.46151	0.25947	-0.12542	O139	O	-0.14605	-0.35420	0.22998
Ca20	Ca	0.24472	-0.27175	0.37505	O80	O	0.46151	0.25881	0.37742	O140	O	-0.14603	-0.35405	-0.26990
Ca21	Ca	-0.25519	0.22824	-0.12504	O81	O	-0.21077	0.01065	-0.12507	O141	O	0.35396	0.14595	0.23004
Ca22	Ca	-0.25507	0.22868	0.37518	O82	O	-0.21135	0.01102	0.37520	O142	O	0.35397	0.14577	-0.27000
Ca23	Ca	-0.25453	-0.27181	-0.12494	O83	O	-0.21041	-0.49086	-0.12502	O143	O	0.35437	-0.35464	0.22885
Ca24	Ca	-0.25449	-0.27220	0.37533	O84	O	-0.21118	-0.49179	0.37520	O144	O	0.35400	-0.35430	-0.27095
Ca25	Ca	-0.24493	-0.22867	0.12482	O85	O	0.28867	0.00820	-0.12480	O145	O	-0.10230	-0.10734	0.01999
Ca26	Ca	-0.24481	-0.22823	-0.37496	O86	O	0.28961	0.00912	0.37496	O146	O	-0.10516	-0.10459	-0.47968
Ca27	Ca	-0.24551	0.27220	0.12467	O87	O	0.28858	-0.48901	-0.12479	O147	O	-0.10497	0.39467	0.02118
Ca28	Ca	-0.24547	0.27181	-0.37506	O88	O	0.28931	-0.48936	0.37497	O148	O	-0.10523	0.39475	-0.47900
Ca29	Ca	0.25463	-0.22811	0.12498	O89	O	0.21039	-0.00911	0.12504	O149	O	0.39472	-0.10520	0.02097
Ca30	Ca	0.25444	-0.22774	-0.37531	O90	O	0.21133	-0.00819	-0.37520	O150	O	0.39523	-0.10518	-0.47884
Ca31	Ca	0.25528	0.27175	0.12495	O91	O	0.21068	0.48937	0.12503	O151	O	0.39480	0.39541	0.02024
Ca32	Ca	0.25508	0.27127	-0.37510	O92	O	0.21142	0.48901	-0.37521	O152	O	0.39779	0.39268	-0.47987
Ge33	Ge	0.00003	-0.25003	-0.00003	O93	O	-0.28865	-0.01102	0.12480	O153	O	0.10389	0.10420	-0.22896
Ti34	Ti	-0.00004	-0.25000	-0.49994	O94	O	-0.28923	-0.01064	-0.37493	O154	O	0.10399	0.10425	0.27119
Ti35	Ti	0.00002	0.24988	-0.00002	O95	O	-0.28882	0.49180	0.12480	O155	O	0.10398	-0.39586	-0.22998
Ti36	Ti	-0.00001	0.25006	-0.49996	O96	O	-0.28959	0.49087	-0.37498	O156	O	0.10400	-0.39521	0.26962
Ti37	Ti	-0.49999	-0.25006	-0.00004	O97	O	-0.14476	0.14404	0.02116	O157	O	-0.39601	0.10476	-0.23044
Ti38	Ti	-0.50002	-0.24988	-0.49998	O98	O	-0.14525	0.14496	-0.47883	O158	O	-0.39594	0.10425	0.27011
Ti39	Ti	-0.49996	0.25000	-0.00006	O99	O	-0.14289	-0.35272	0.02025	O159	O	-0.39592	-0.39570	-0.22887
Ge40	Ge	-0.50003	0.25004	-0.49997	O100	O	-0.14562	-0.35539	-0.47963	O160	O	-0.39604	-0.39575	0.27117
Ti41	Ti	0.00016	-0.25035	-0.24894	O101	O	0.35427	0.14467	0.02022					
Ti42	Ti	-0.00020	-0.24967	0.24895	O102	O	0.35708	0.14731	-0.47977					
Ti43	Ti	0.00003	0.24981	-0.24993	O103	O	0.35469	-0.35495	0.02105					
Ti44	Ti	-0.00004	0.25011	0.24991	O104	O	0.35535	-0.35598	-0.47875					
Ti45	Ti	-0.50003	-0.24981	-0.25007	O105	O	0.14603	-0.14577	-0.23000					
Ti46	Ti	-0.49996	-0.25011	0.25009	O106	O	0.14604	-0.14594	0.26996					
Ti47	Ti	-0.50016	0.25036	-0.25106	O107	O	0.14600	0.35431	-0.22905					
Ti48	Ti	-0.49981	0.24968	0.25105	O108	O	0.14563	0.35464	0.27115					
Ti49	Ti	-0.24898	-0.00126	-0.00024	O109	O	-0.35427	-0.14540	-0.22892					
Ti50	Ti	-0.25102	0.00126	-0.49976	O110	O	-0.35406	-0.14562	0.27110					
Ti51	Ti	-0.24869	-0.49905	-0.00024	O111	O	-0.35397	0.35406	-0.23010					
Ti52	Ti	-0.25131	-0.50094	-0.49976	O112	O	-0.35395	0.35421	0.27002					
Ti53	Ti	0.24872	-0.00104	0.00026	O113	O	0.10477	0.10519	-0.02116					
Ti54	Ti	0.25128	0.00105	-0.50026	O114	O	0.10528	0.10521	0.47903					
Ti55	Ti	0.24892	-0.49877	0.00022	O115	O	0.10221	-0.39267	-0.02013					
Ti56	Ti	0.25108	-0.50123	-0.50022	O116	O	0.10520	-0.39540	0.47976					
Ti57	Ti	-0.25000	0.00000	-0.25000	O117	O	-0.39484	0.10459	-0.02032					
Ti58	Ti	-0.25000	0.00000	0.25000	O118	O	-0.39770	0.10735	0.48001					
Ti59	Ti	-0.25000	-0.50000	-0.25000	O119	O	-0.39477	-0.39475	-0.02100					
Ti60	Ti	-0.25000	-0.50000	0.25000	O120	O	-0.39503	-0.39467	0.47881					

Table SI4. Atomic coordinates obtained from the DFT calculation for the sample CTG at P = 0.206 GPa.

Atom label	Atom type	*x*	*y*	*z*	Atom label	Atom type	*x*	*y*	*z*	Atom label	Atom type	*x*	*y*	*z*
Ca1	Ca	-0.00490	0.02147	0.12512	Ti61	Ti	0.25000	0.00000	-0.25000	O121	O	-0.10402	-0.10421	0.22987
Ca2	Ca	-0.00443	0.02135	-0.37492	Ti62	Ti	0.25000	0.00000	0.25000	O122	O	-0.10395	-0.10472	-0.26957
Ca3	Ca	-0.00469	-0.47711	0.12429	Ti63	Ti	0.25000	-0.50000	-0.25000	O123	O	-0.10393	0.39579	0.22881
Ca4	Ca	-0.00415	-0.47803	-0.37537	Ti64	Ti	0.25000	-0.50000	0.25000	O124	O	-0.10404	0.39574	-0.27116
Ca5	Ca	0.49585	0.02205	0.12451	O65	O	0.04035	0.23965	0.12496	O125	O	0.39605	-0.10421	0.22879
Ca6	Ca	0.49538	0.02291	-0.37564	O66	O	0.03998	0.23990	-0.37501	O126	O	0.39615	-0.10416	-0.27107
Ca7	Ca	0.49552	-0.47861	0.12509	O67	O	0.03852	-0.25880	0.12258	O127	O	0.39603	0.39525	0.23037
Ca8	Ca	0.49515	-0.47836	-0.37481	O68	O	0.03853	-0.25946	-0.37458	O128	O	0.39605	0.39590	-0.27004
Ca9	Ca	0.00462	-0.02290	-0.12436	O69	O	-0.46141	0.24043	0.12538	O129	O	0.14294	-0.14733	-0.02025
Ca10	Ca	0.00415	-0.02204	0.37549	O70	O	-0.46155	0.24115	-0.37739	O130	O	0.14576	-0.14469	0.47976
Ca11	Ca	0.00485	0.47836	-0.12519	O71	O	-0.46001	-0.25999	0.12498	O131	O	0.14468	0.35596	-0.02127
Ca12	Ca	0.00448	0.47861	0.37491	O72	O	-0.45971	-0.26021	-0.37498	O132	O	0.14535	0.35493	0.47893
Ca13	Ca	-0.49557	-0.02134	-0.12508	O73	O	-0.03845	-0.24115	-0.12261	O133	O	-0.35471	-0.14498	-0.02119
Ca14	Ca	-0.49510	-0.02146	0.37488	O74	O	-0.03859	-0.24042	0.37462	O134	O	-0.35522	-0.14405	0.47881
Ca15	Ca	-0.49585	0.47803	-0.12463	O75	O	-0.04029	0.26022	-0.12502	O135	O	-0.35435	0.35537	-0.02038
Ca16	Ca	-0.49531	0.47712	0.37571	O76	O	-0.03999	0.26000	0.37502	O136	O	-0.35709	0.35271	0.47974
Ca17	Ca	0.24557	0.22773	-0.12469	O77	O	0.46002	-0.23989	-0.12499	O137	O	-0.14598	0.14566	0.22887
Ca18	Ca	0.24537	0.22810	0.37502	O78	O	0.45965	-0.23964	0.37504	O138	O	-0.14576	0.14544	-0.27110
Ca19	Ca	0.24491	-0.27128	-0.12491	O79	O	0.46147	0.25947	-0.12542	O139	O	-0.14609	-0.35417	0.22996
Ca20	Ca	0.24471	-0.27177	0.37505	O80	O	0.46148	0.25881	0.37742	O140	O	-0.14607	-0.35401	-0.26992
Ca21	Ca	-0.25519	0.22822	-0.12504	O81	O	-0.21072	0.01065	-0.12507	O141	O	0.35393	0.14598	0.23002
Ca22	Ca	-0.25507	0.22866	0.37518	O82	O	-0.21131	0.01103	0.37520	O142	O	0.35394	0.14580	-0.27002
Ca23	Ca	-0.25453	-0.27183	-0.12495	O83	O	-0.21036	-0.49086	-0.12502	O143	O	0.35434	-0.35461	0.22883
Ca24	Ca	-0.25449	-0.27222	0.37534	O84	O	-0.21114	-0.49179	0.37520	O144	O	0.35396	-0.35427	-0.27097
Ca25	Ca	-0.24493	-0.22865	0.12482	O85	O	0.28872	0.00819	-0.12480	O145	O	-0.10227	-0.10732	0.02001
Ca26	Ca	-0.24481	-0.22821	-0.37496	O86	O	0.28965	0.00912	0.37496	O146	O	-0.10513	-0.10456	-0.47966
Ca27	Ca	-0.24551	0.27222	0.12466	O87	O	0.28862	-0.48900	-0.12479	O147	O	-0.10493	0.39470	0.02121
Ca28	Ca	-0.24547	0.27183	-0.37505	O88	O	0.28936	-0.48935	0.37497	O148	O	-0.10520	0.39478	-0.47898
Ca29	Ca	0.25463	-0.22809	0.12498	O89	O	0.21035	-0.00911	0.12504	O149	O	0.39475	-0.10518	0.02099
Ca30	Ca	0.25443	-0.22772	-0.37531	O90	O	0.21128	-0.00819	-0.37520	O150	O	0.39526	-0.10516	-0.47882
Ca31	Ca	0.25529	0.27177	0.12495	O91	O	0.21064	0.48936	0.12503	O151	O	0.39483	0.39544	0.02026
Ca32	Ca	0.25509	0.27129	-0.37509	O92	O	0.21138	0.48901	-0.37521	O152	O	0.39782	0.39270	-0.47986
Ge33	Ge	0.00003	-0.25003	-0.00003	O93	O	-0.28869	-0.01102	0.12480	O153	O	0.10386	0.10417	-0.22893
Ti34	Ti	-0.00004	-0.25000	-0.49994	O94	O	-0.28928	-0.01065	-0.37493	O154	O	0.10395	0.10421	0.27121
Ti35	Ti	0.00002	0.24989	-0.00001	O95	O	-0.28886	0.49180	0.12480	O155	O	0.10395	-0.39589	-0.22996
Ti36	Ti	-0.00000	0.25006	-0.49996	O96	O	-0.28964	0.49087	-0.37498	O156	O	0.10397	-0.39524	0.26963
Ti37	Ti	-0.50000	-0.25006	-0.00004	O97	O	-0.14479	0.14406	0.02119	O157	O	-0.39605	0.10473	-0.23043
Ti38	Ti	-0.50002	-0.24988	-0.49999	O98	O	-0.14529	0.14499	-0.47881	O158	O	-0.39598	0.10421	0.27013
Ti39	Ti	-0.49996	0.25000	-0.00006	O99	O	-0.14291	-0.35270	0.02026	O159	O	-0.39596	-0.39573	-0.22884
Ge40	Ge	-0.50003	0.25004	-0.49997	O100	O	-0.14566	-0.35537	-0.47962	O160	O	-0.39607	-0.39578	0.27119
Ti41	Ti	0.00016	-0.25035	-0.24894	O101	O	0.35424	0.14470	0.02024					
Ti42	Ti	-0.00019	-0.24967	0.24895	O102	O	0.35705	0.14734	-0.47975					
Ti43	Ti	0.00003	0.24981	-0.24993	O103	O	0.35465	-0.35492	0.02107					
Ti44	Ti	-0.00004	0.25011	0.24991	O104	O	0.35532	-0.35596	-0.47873					
Ti45	Ti	-0.50003	-0.24981	-0.25007	O105	O	0.14606	-0.14580	-0.22998					
Ti46	Ti	-0.49996	-0.25011	0.25009	O106	O	0.14607	-0.14597	0.26998					
Ti47	Ti	-0.50016	0.25036	-0.25106	O107	O	0.14604	0.35428	-0.22903					
Ti48	Ti	-0.49981	0.24968	0.25105	O108	O	0.14566	0.35461	0.27117					
Ti49	Ti	-0.24897	-0.00126	-0.00024	O109	O	-0.35424	-0.14543	-0.22890					
Ti50	Ti	-0.25103	0.00127	-0.49976	O110	O	-0.35402	-0.14565	0.27113					
Ti51	Ti	-0.24869	-0.49904	-0.00024	O111	O	-0.35394	0.35402	-0.23008					
Ti52	Ti	-0.25131	-0.50095	-0.49976	O112	O	-0.35391	0.35418	0.27004					
Ti53	Ti	0.24872	-0.00104	0.00026	O113	O	0.10474	0.10516	-0.02118					
Ti54	Ti	0.25128	0.00105	-0.50026	O114	O	0.10525	0.10518	0.47901					
Ti55	Ti	0.24891	-0.49876	0.00022	O115	O	0.10218	-0.39270	-0.02014					
Ti56	Ti	0.25109	-0.50123	-0.50022	O116	O	0.10517	-0.39543	0.47974					
Ti57	Ti	-0.25000	0.00000	-0.25000	O117	O	-0.39487	0.10457	-0.02034					
Ti58	Ti	-0.25000	0.00000	0.25000	O118	O	-0.39773	0.10733	0.47999					
Ti59	Ti	-0.25000	-0.50000	-0.25000	O119	O	-0.39480	-0.39477	-0.02102					
Ti60	Ti	-0.25000	-0.50000	0.25000	O120	O	-0.39507	-0.39470	0.47879					

Table SI5. Atomic coordinates obtained from the DFT calculation for the sample CTG at P = 1.623 GPa.

Atom label	Atom type	*x*	*y*	*z*
Ca1	Ca	-0.00494	0.02161	0.12511
Ca2	Ca	-0.00446	0.02145	-0.37493
Ca3	Ca	-0.00471	-0.47700	0.12433
Ca4	Ca	-0.00416	-0.47787	-0.37538
Ca5	Ca	0.49583	0.02221	0.12449
Ca6	Ca	0.49536	0.02302	-0.37561
Ca7	Ca	0.49547	-0.47850	0.12507
Ca8	Ca	0.49512	-0.47821	-0.37482
Ca9	Ca	0.00464	-0.02301	-0.12439
Ca10	Ca	0.00417	-0.02221	0.37551
Ca11	Ca	0.00488	0.47821	-0.12518
Ca12	Ca	0.00453	0.47850	0.37493
Ca13	Ca	-0.49554	-0.02145	-0.12507
Ca14	Ca	-0.49506	-0.02161	0.37489
Ca15	Ca	-0.49584	0.47788	-0.12463
Ca16	Ca	-0.49529	0.47701	0.37567
Ca17	Ca	0.24557	0.22758	-0.12468
Ca18	Ca	0.24536	0.22795	0.37499
Ca19	Ca	0.24491	-0.27140	-0.12492
Ca20	Ca	0.24466	-0.27191	0.37507
Ca21	Ca	-0.25525	0.22808	-0.12501
Ca22	Ca	-0.25508	0.22854	0.37518
Ca23	Ca	-0.25455	-0.27198	-0.12497
Ca24	Ca	-0.25447	-0.27236	0.37535
Ca25	Ca	-0.24492	-0.22854	0.12483
Ca26	Ca	-0.24476	-0.22808	-0.37499
Ca27	Ca	-0.24553	0.27237	0.12465
Ca28	Ca	-0.24545	0.27199	-0.37503
Ca29	Ca	0.25464	-0.22794	0.12501
Ca30	Ca	0.25443	-0.22758	-0.37532
Ca31	Ca	0.25534	0.27191	0.12493
Ca32	Ca	0.25509	0.27140	-0.37508
Ge33	Ge	0.00003	-0.25003	-0.00003
Ti34	Ti	-0.00003	-0.25001	-0.49994
Ti35	Ti	-0.00000	0.24990	0.00001
Ti36	Ti	0.00003	0.25004	-0.49995
Ti37	Ti	-0.50003	-0.25004	-0.00005
Ti38	Ti	-0.50000	-0.24990	-0.50001
Ti39	Ti	-0.49997	0.25002	-0.00006
Ge40	Ge	-0.50003	0.25003	-0.49997
Ti41	Ti	0.00015	-0.25036	-0.24891
Ti42	Ti	-0.00018	-0.24967	0.24893
Ti43	Ti	0.00002	0.24981	-0.24992
Ti44	Ti	-0.00005	0.25010	0.24992
Ti45	Ti	-0.50002	-0.24981	-0.25009
Ti46	Ti	-0.49995	-0.25010	0.25008
Ti47	Ti	-0.50015	0.25036	-0.25109
Ti48	Ti	-0.49982	0.24967	0.25107
Ti49	Ti	-0.24895	-0.00129	-0.00024
Ti50	Ti	-0.25105	0.00130	-0.49976
Ti51	Ti	-0.24869	-0.49899	-0.00023
Ti52	Ti	-0.25131	-0.50100	-0.49977
Ti53	Ti	0.24868	-0.00106	0.00025
Ti54	Ti	0.25132	0.00107	-0.50025
Ti55	Ti	0.24886	-0.49874	0.00021
Ti56	Ti	0.25114	-0.50126	-0.50021
Ti57	Ti	-0.25000	0.00000	-0.25000
Ti58	Ti	-0.25000	0.00000	0.25000
Ti59	Ti	-0.25000	-0.50000	-0.25000
Ti60	Ti	-0.25000	-0.50000	0.25000
Ti61	Ti	0.25000	0.00000	-0.25000
Ti62	Ti	0.25000	0.00000	0.25000
Ti63	Ti	0.25000	-0.50000	-0.25000
Ti64	Ti	0.25000	-0.50000	0.25000
O65	O	0.04069	0.23962	0.12496
O66	O	0.04029	0.23987	-0.37501
O67	O	0.03874	-0.25877	0.12256
O68	O	0.03880	-0.25945	-0.37457
O69	O	-0.46115	0.24044	0.12538
O70	O	-0.46133	0.24119	-0.37741
O71	O	-0.45970	-0.26004	0.12498
O72	O	-0.45939	-0.26023	-0.37499
O73	O	-0.03867	-0.24119	-0.12259
O74	O	-0.03885	-0.24044	0.37462
O75	O	-0.04061	0.26024	-0.12501
O76	O	-0.04030	0.26004	0.37502
O77	O	0.45971	-0.23986	-0.12499
O78	O	0.45931	-0.23961	0.37504
O79	O	0.46120	0.25945	-0.12543
O80	O	0.46126	0.25877	0.37744
O81	O	-0.21040	0.01069	-0.12508
O82	O	-0.21100	0.01107	0.37520
O83	O	-0.21005	-0.49087	-0.12503
O84	O	-0.21083	-0.49181	0.37521
O85	O	0.28903	0.00817	-0.12480
O86	O	0.28996	0.00913	0.37495
O87	O	0.28893	-0.48897	-0.12479
O88	O	0.28967	-0.48932	0.37496
O89	O	0.21004	-0.00912	0.12505
O90	O	0.21097	-0.00817	-0.37520
O91	O	0.21033	0.48932	0.12504
O92	O	0.21107	0.48897	-0.37521
O93	O	-0.28900	-0.01106	0.12480
O94	O	-0.28960	-0.01069	-0.37492
O95	O	-0.28917	0.49182	0.12479
O96	O	-0.28995	0.49087	-0.37497
O97	O	-0.14500	0.14424	0.02136
O98	O	-0.14552	0.14521	-0.47866
O99	O	-0.14308	-0.35253	0.02036
O100	O	-0.14589	-0.35518	-0.47949
O101	O	0.35402	0.14488	0.02037
O102	O	0.35687	0.14752	-0.47965
O103	O	0.35443	-0.35472	0.02123
O104	O	0.35510	-0.35577	-0.47856
O105	O	0.14630	-0.14600	-0.22985
O106	O	0.14633	-0.14620	0.27011
O107	O	0.14629	0.35405	-0.22888
O108	O	0.14589	0.35442	0.27132
O109	O	-0.35401	-0.14563	-0.22874
O110	O	-0.35378	-0.14587	0.27129
O111	O	-0.35368	0.35380	-0.22995
O112	O	-0.35367	0.35398	0.27018
O113	O	0.10451	0.10497	-0.02134
O114	O	0.10501	0.10498	0.47885
O115	O	0.10198	-0.39285	-0.02025
O116	O	0.10496	-0.39562	0.47961
O117	O	-0.39507	0.10437	-0.02047
O118	O	-0.39794	0.10718	0.47989
O119	O	-0.39502	-0.39498	-0.02118
O120	O	-0.39531	-0.39488	0.47863
O121	O	-0.10379	-0.10399	0.22973
O122	O	-0.10371	-0.10451	-0.26971
O123	O	-0.10369	0.39599	0.22865
O124	O	-0.10380	0.39595	-0.27132
O125	O	0.39630	-0.10399	0.22863
O126	O	0.39638	-0.10395	-0.27122
O127	O	0.39628	0.39546	0.23024
O128	O	0.39629	0.39612	-0.27017
O129	O	0.14313	-0.14751	-0.02035
O130	O	0.14598	-0.14487	0.47963
O131	O	0.14490	0.35577	-0.02144
O132	O	0.14557	0.35473	0.47877
O133	O	-0.35448	-0.14520	-0.02134
O134	O	-0.35500	-0.14424	0.47864
O135	O	-0.35411	0.35518	-0.02051
O136	O	-0.35692	0.35254	0.47964
O137	O	-0.14622	0.14588	0.22871
O138	O	-0.14599	0.14563	-0.27126
O139	O	-0.14633	-0.35397	0.22982
O140	O	-0.14632	-0.35379	-0.27005
O141	O	0.35367	0.14621	0.22989
O142	O	0.35370	0.14600	-0.27015
O143	O	0.35410	-0.35441	0.22868
O144	O	0.35371	-0.35405	-0.27112
O145	O	-0.10206	-0.10717	0.02011
O146	O	-0.10493	-0.10436	-0.47953
O147	O	-0.10469	0.39488	0.02137
O148	O	-0.10498	0.39499	-0.47882
O149	O	0.39499	-0.10498	0.02115
O150	O	0.39549	-0.10496	-0.47866
O151	O	0.39504	0.39563	0.02039
O152	O	0.39802	0.39285	-0.47975
O153	O	0.10362	0.10396	-0.22878
O154	O	0.10370	0.10400	0.27137
O155	O	0.10371	-0.39611	-0.22983
O156	O	0.10372	-0.39546	0.26976
O157	O	-0.39629	0.10451	-0.23029
O158	O	-0.39621	0.10400	0.27027
O159	O	-0.39620	-0.39594	-0.22868
O160	O	-0.39631	-0.39599	0.27135

Table SI6. Atomic coordinates obtained from the DFT calculation for the sample CTG at P = 2.530 GPa.

Atom label	Atom type	*x*	*y*	*z*	Atom label	Atom type	*x*	*y*	*z*	Atom label	Atom type	*x*	*y*	*z*
Ca1	Ca	-0.00496	0.02171	0.12510	Ti61	Ti	0.25000	0.00000	-0.25000	O121	O	-0.10364	-0.10387	0.22965
Ca2	Ca	-0.00448	0.02153	-0.37493	Ti62	Ti	0.25000	0.00000	0.25000	O122	O	-0.10356	-0.10438	-0.26979
Ca3	Ca	-0.00473	-0.47693	0.12435	Ti63	Ti	0.25000	-0.50000	-0.25000	O123	O	-0.10355	0.39612	0.22855
Ca4	Ca	-0.00417	-0.47777	-0.37538	Ti64	Ti	0.25000	-0.50000	0.25000	O124	O	-0.10364	0.39608	-0.27142
Ca5	Ca	0.49582	0.02232	0.12449	O65	O	0.04090	0.23960	0.12496	O125	O	0.39645	-0.10387	0.22853
Ca6	Ca	0.49535	0.02309	-0.37559	O66	O	0.04048	0.23985	-0.37501	O126	O	0.39653	-0.10382	-0.27131
Ca7	Ca	0.49545	-0.47842	0.12506	O67	O	0.03889	-0.25875	0.12256	O127	O	0.39642	0.39559	0.23015
Ca8	Ca	0.49510	-0.47811	-0.37483	O68	O	0.03897	-0.25944	-0.37457	O128	O	0.39643	0.39625	-0.27025
Ca9	Ca	0.00465	-0.02309	-0.12441	O69	O	-0.46099	0.24046	0.12539	O129	O	0.14324	-0.14762	-0.02041
Ca10	Ca	0.00418	-0.02232	0.37551	O70	O	-0.46119	0.24122	-0.37741	O130	O	0.14612	-0.14499	0.47955
Ca11	Ca	0.00490	0.47811	-0.12517	O71	O	-0.45951	-0.26006	0.12499	O131	O	0.14505	0.35565	-0.02154
Ca12	Ca	0.00456	0.47842	0.37494	O72	O	-0.45919	-0.26025	-0.37499	O132	O	0.14571	0.35460	0.47868
Ca13	Ca	-0.49552	-0.02153	-0.12507	O73	O	-0.03881	-0.24121	-0.12259	O133	O	-0.35434	-0.14534	-0.02143
Ca14	Ca	-0.49504	-0.02170	0.37490	O74	O	-0.03901	-0.24045	0.37461	O134	O	-0.35486	-0.14435	0.47854
Ca15	Ca	-0.49583	0.47778	-0.12462	O75	O	-0.04081	0.26025	-0.12501	O135	O	-0.35397	0.35506	-0.02059
Ca16	Ca	-0.49527	0.47694	0.37565	O76	O	-0.04049	0.26007	0.37501	O136	O	-0.35681	0.35243	0.47958
Ca17	Ca	0.24557	0.22749	-0.12467	O77	O	0.45952	-0.23984	-0.12499	O137	O	-0.14637	0.14602	0.22862
Ca18	Ca	0.24536	0.22785	0.37498	O78	O	0.45910	-0.23959	0.37504	O138	O	-0.14614	0.14575	-0.27135
Ca19	Ca	0.24491	-0.27148	-0.12493	O79	O	0.46102	0.25945	-0.12543	O139	O	-0.14647	-0.35385	0.22974
Ca20	Ca	0.24463	-0.27201	0.37508	O80	O	0.46111	0.25875	0.37744	O140	O	-0.14648	-0.35365	-0.27014
Ca21	Ca	-0.25527	0.22799	-0.12499	O81	O	-0.21021	0.01072	-0.12508	O141	O	0.35352	0.14634	0.22980
Ca22	Ca	-0.25509	0.22846	0.37517	O82	O	-0.21081	0.01109	0.37520	O142	O	0.35355	0.14613	-0.27024
Ca23	Ca	-0.25456	-0.27209	-0.12499	O83	O	-0.20986	-0.49086	-0.12504	O143	O	0.35396	-0.35429	0.22860
Ca24	Ca	-0.25446	-0.27246	0.37536	O84	O	-0.21064	-0.49182	0.37521	O144	O	0.35356	-0.35391	-0.27121
Ca25	Ca	-0.24491	-0.22846	0.12483	O85	O	0.28922	0.00817	-0.12479	O145	O	-0.10193	-0.10707	0.02018
Ca26	Ca	-0.24473	-0.22798	-0.37501	O86	O	0.29015	0.00914	0.37495	O146	O	-0.10480	-0.10424	-0.47946
Ca27	Ca	-0.24555	0.27246	0.12464	O87	O	0.28912	-0.48895	-0.12479	O147	O	-0.10455	0.39500	0.02147
Ca28	Ca	-0.24544	0.27209	-0.37501	O88	O	0.28986	-0.48930	0.37496	O148	O	-0.10484	0.39512	-0.47873
Ca29	Ca	0.25464	-0.22785	0.12502	O89	O	0.20985	-0.00913	0.12505	O149	O	0.39514	-0.10485	0.02125
Ca30	Ca	0.25442	-0.22749	-0.37533	O90	O	0.21078	-0.00816	-0.37521	O150	O	0.39563	-0.10484	-0.47856
Ca31	Ca	0.25537	0.27201	0.12492	O91	O	0.21014	0.48931	0.12504	O151	O	0.39517	0.39575	0.02047
Ca32	Ca	0.25509	0.27149	-0.37507	O92	O	0.21088	0.48896	-0.37521	O152	O	0.39814	0.39295	-0.47969
Ge33	Ge	0.00003	-0.25003	-0.00003	O93	O	-0.28919	-0.01108	0.12480	O153	O	0.10347	0.10383	-0.22869
Ti34	Ti	-0.00002	-0.25002	-0.49994	O94	O	-0.28979	-0.01071	-0.37492	O154	O	0.10355	0.10387	0.27147
Ti35	Ti	-0.00001	0.24991	0.00002	O95	O	-0.28936	0.49182	0.12479	O155	O	0.10357	-0.39625	-0.22975
Ti36	Ti	0.00004	0.25004	-0.49995	O96	O	-0.29014	0.49087	-0.37496	O156	O	0.10358	-0.39559	0.26985
Ti37	Ti	-0.50004	-0.25003	-0.00005	O97	O	-0.14514	0.14436	0.02146	O157	O	-0.39644	0.10438	-0.23021
Ti38	Ti	-0.49999	-0.24990	-0.50002	O98	O	-0.14566	0.14534	-0.47856	O158	O	-0.39636	0.10387	0.27035
Ti39	Ti	-0.49998	0.25002	-0.00006	O99	O	-0.14319	-0.35242	0.02042	O159	O	-0.39636	-0.39607	-0.22858
Ge40	Ge	-0.50003	0.25003	-0.49997	O100	O	-0.14603	-0.35506	-0.47941	O160	O	-0.39645	-0.39611	0.27145
Ti41	Ti	0.00014	-0.25036	-0.24889	O101	O	0.35388	0.14499	0.02045					
Ti42	Ti	-0.00017	-0.24967	0.24892	O102	O	0.35676	0.14763	-0.47958					
Ti43	Ti	0.00002	0.24981	-0.24991	O103	O	0.35429	-0.35460	0.02132					
Ti44	Ti	-0.00005	0.25010	0.24993	O104	O	0.35495	-0.35564	-0.47846					
Ti45	Ti	-0.50002	-0.24981	-0.25009	O105	O	0.14645	-0.14612	-0.22976					
Ti46	Ti	-0.49995	-0.25010	0.25007	O106	O	0.14648	-0.14633	0.27020					
Ti47	Ti	-0.50014	0.25036	-0.25111	O107	O	0.14644	0.35392	-0.22879					
Ti48	Ti	-0.49983	0.24968	0.25109	O108	O	0.14604	0.35430	0.27140					
Ti49	Ti	-0.24894	-0.00131	-0.00024	O109	O	-0.35386	-0.14575	-0.22865					
Ti50	Ti	-0.25106	0.00131	-0.49976	O110	O	-0.35363	-0.14601	0.27138					
Ti51	Ti	-0.24869	-0.49897	-0.00023	O111	O	-0.35353	0.35366	-0.22986					
Ti52	Ti	-0.25131	-0.50102	-0.49977	O112	O	-0.35353	0.35386	0.27026					
Ti53	Ti	0.24867	-0.00107	0.00025	O113	O	0.10437	0.10484	-0.02144					
Ti54	Ti	0.25133	0.00108	-0.50025	O114	O	0.10486	0.10486	0.47876					
Ti55	Ti	0.24883	-0.49873	0.00021	O115	O	0.10186	-0.39295	-0.02031					
Ti56	Ti	0.25116	-0.50127	-0.50021	O116	O	0.10483	-0.39574	0.47954					
Ti57	Ti	-0.25000	0.00000	-0.25000	O117	O	-0.39520	0.10425	-0.02054					
Ti58	Ti	-0.25000	0.00000	0.25000	O118	O	-0.39807	0.10708	0.47982					
Ti59	Ti	-0.25000	-0.50000	-0.25000	O119	O	-0.39516	-0.39511	-0.02127					
Ti60	Ti	-0.25000	-0.50000	0.25000	O120	O	-0.39545	-0.39500	0.47853					

Table SI7. Atomic coordinates obtained from the DFT calculation for the sample CTG at P = 3.032 GPa.

Atom label	Atom type	*x*	*y*	*z*	Atom label	Atom type	*x*	*y*	*z*	Atom label	Atom type	*x*	*y*	*z*
Ca1	Ca	-0.00498	0.02177	0.12510	Ti61	Ti	0.25000	0.00000	-0.25000	O121	O	-0.10357	-0.10380	0.22961
Ca2	Ca	-0.00450	0.02158	-0.37493	Ti62	Ti	0.25000	0.00000	0.25000	O122	O	-0.10348	-0.10431	-0.26984
Ca3	Ca	-0.00474	-0.47689	0.12437	Ti63	Ti	0.25000	-0.50000	-0.25000	O123	O	-0.10348	0.39618	0.22850
Ca4	Ca	-0.00417	-0.47772	-0.37538	Ti64	Ti	0.25000	-0.50000	0.25000	O124	O	-0.10356	0.39614	-0.27147
Ca5	Ca	0.49581	0.02238	0.12448	O65	O	0.04102	0.23959	0.12497	O125	O	0.39653	-0.10380	0.22848
Ca6	Ca	0.49534	0.02313	-0.37558	O66	O	0.04058	0.23984	-0.37501	O126	O	0.39660	-0.10376	-0.27136
Ca7	Ca	0.49543	-0.47837	0.12506	O67	O	0.03896	-0.25874	0.12255	O127	O	0.39650	0.39567	0.23011
Ca8	Ca	0.49508	-0.47805	-0.37484	O68	O	0.03906	-0.25944	-0.37457	O128	O	0.39651	0.39633	-0.27029
Ca9	Ca	0.00466	-0.02313	-0.12442	O69	O	-0.46090	0.24047	0.12539	O129	O	0.14330	-0.14768	-0.02045
Ca10	Ca	0.00419	-0.02238	0.37552	O70	O	-0.46112	0.24123	-0.37742	O130	O	0.14620	-0.14505	0.47951
Ca11	Ca	0.00492	0.47805	-0.12516	O71	O	-0.45941	-0.26008	0.12499	O131	O	0.14512	0.35558	-0.02159
Ca12	Ca	0.00457	0.47837	0.37494	O72	O	-0.45908	-0.26025	-0.37499	O132	O	0.14578	0.35454	0.47863
Ca13	Ca	-0.49550	-0.02157	-0.12507	O73	O	-0.03888	-0.24122	-0.12258	O133	O	-0.35426	-0.14540	-0.02149
Ca14	Ca	-0.49502	-0.02176	0.37490	O74	O	-0.03910	-0.24046	0.37461	O134	O	-0.35478	-0.14442	0.47848
Ca15	Ca	-0.49583	0.47772	-0.12462	O75	O	-0.04092	0.26026	-0.12501	O135	O	-0.35389	0.35500	-0.02063
Ca16	Ca	-0.49526	0.47689	0.37563	O76	O	-0.04059	0.26009	0.37501	O136	O	-0.35675	0.35237	0.47954
Ca17	Ca	0.24557	0.22743	-0.12466	O77	O	0.45942	-0.23983	-0.12499	O137	O	-0.14645	0.14609	0.22857
Ca18	Ca	0.24535	0.22779	0.37497	O78	O	0.45898	-0.23958	0.37503	O138	O	-0.14621	0.14582	-0.27140
Ca19	Ca	0.24491	-0.27153	-0.12493	O79	O	0.46093	0.25944	-0.12543	O139	O	-0.14655	-0.35379	0.22969
Ca20	Ca	0.24462	-0.27207	0.37508	O80	O	0.46104	0.25874	0.37745	O140	O	-0.14656	-0.35358	-0.27019
Ca21	Ca	-0.25529	0.22793	-0.12499	O81	O	-0.21010	0.01073	-0.12508	O141	O	0.35344	0.14642	0.22975
Ca22	Ca	-0.25510	0.22842	0.37517	O82	O	-0.21070	0.01110	0.37520	O142	O	0.35347	0.14619	-0.27028
Ca23	Ca	-0.25456	-0.27215	-0.12500	O83	O	-0.20976	-0.49087	-0.12504	O143	O	0.35388	-0.35423	0.22855
Ca24	Ca	-0.25445	-0.27251	0.37536	O84	O	-0.21053	-0.49182	0.37521	O144	O	0.35348	-0.35384	-0.27126
Ca25	Ca	-0.24490	-0.22841	0.12483	O85	O	0.28933	0.00816	-0.12479	O145	O	-0.10186	-0.10702	0.02021
Ca26	Ca	-0.24471	-0.22793	-0.37501	O86	O	0.29025	0.00914	0.37494	O146	O	-0.10473	-0.10418	-0.47941
Ca27	Ca	-0.24555	0.27252	0.12464	O87	O	0.28922	-0.48894	-0.12479	O147	O	-0.10447	0.39506	0.02152
Ca28	Ca	-0.24544	0.27216	-0.37500	O88	O	0.28996	-0.48929	0.37496	O148	O	-0.10476	0.39519	-0.47868
Ca29	Ca	0.25464	-0.22779	0.12503	O89	O	0.20975	-0.00913	0.12506	O149	O	0.39522	-0.10478	0.02130
Ca30	Ca	0.25442	-0.22743	-0.37534	O90	O	0.21067	-0.00816	-0.37521	O150	O	0.39570	-0.10478	-0.47851
Ca31	Ca	0.25538	0.27207	0.12492	O91	O	0.21004	0.48929	0.12504	O151	O	0.39524	0.39581	0.02051
Ca32	Ca	0.25509	0.27153	-0.37507	O92	O	0.21078	0.48895	-0.37521	O152	O	0.39821	0.39300	-0.47966
Ge33	Ge	0.00003	-0.25003	-0.00002	O93	O	-0.28930	-0.01109	0.12480	O153	O	0.10340	0.10376	-0.22864
Ti34	Ti	-0.00002	-0.25002	-0.49994	O94	O	-0.28990	-0.01073	-0.37492	O154	O	0.10347	0.10381	0.27152
Ti35	Ti	-0.00001	0.24991	0.00002	O95	O	-0.28947	0.49183	0.12479	O155	O	0.10349	-0.39632	-0.22971
Ti36	Ti	0.00004	0.25003	-0.49995	O96	O	-0.29024	0.49087	-0.37496	O156	O	0.10350	-0.39566	0.26989
Ti37	Ti	-0.50004	-0.25003	-0.00005	O97	O	-0.14522	0.14443	0.02152	O157	O	-0.39652	0.10431	-0.23016
Ti38	Ti	-0.49999	-0.24991	-0.50002	O98	O	-0.14574	0.14541	-0.47851	O158	O	-0.39643	0.10380	0.27039
Ti39	Ti	-0.49998	0.25002	-0.00006	O99	O	-0.14325	-0.35237	0.02046	O159	O	-0.39644	-0.39614	-0.22853
Ge40	Ge	-0.50003	0.25003	-0.49997	O100	O	-0.14611	-0.35500	-0.47937	O160	O	-0.39653	-0.39618	0.27150
Ti41	Ti	0.00013	-0.25036	-0.24889	O101	O	0.35380	0.14505	0.02049					
Ti42	Ti	-0.00017	-0.24967	0.24891	O102	O	0.35670	0.14769	-0.47955					
Ti43	Ti	0.00002	0.24981	-0.24991	O103	O	0.35422	-0.35453	0.02138					
Ti44	Ti	-0.00005	0.25010	0.24993	O104	O	0.35488	-0.35558	-0.47841					
Ti45	Ti	-0.50002	-0.24981	-0.25009	O105	O	0.14653	-0.14619	-0.22972					
Ti46	Ti	-0.49995	-0.25010	0.25007	O106	O	0.14657	-0.14641	0.27025					
Ti47	Ti	-0.50013	0.25036	-0.25111	O107	O	0.14652	0.35384	-0.22874					
Ti48	Ti	-0.49984	0.24968	0.25109	O108	O	0.14612	0.35424	0.27145					
Ti49	Ti	-0.24894	-0.00131	-0.00024	O109	O	-0.35379	-0.14581	-0.22860					
Ti50	Ti	-0.25106	0.00132	-0.49976	O110	O	-0.35355	-0.14608	0.27143					
Ti51	Ti	-0.24869	-0.49896	-0.00022	O111	O	-0.35344	0.35358	-0.22981					
Ti52	Ti	-0.25131	-0.50104	-0.49977	O112	O	-0.35345	0.35380	0.27031					
Ti53	Ti	0.24866	-0.00108	0.00025	O113	O	0.10430	0.10478	-0.02149					
Ti54	Ti	0.25134	0.00108	-0.50025	O114	O	0.10478	0.10479	0.47870					
Ti55	Ti	0.24882	-0.49872	0.00020	O115	O	0.10179	-0.39300	-0.02034					
Ti56	Ti	0.25118	-0.50127	-0.50020	O116	O	0.10476	-0.39581	0.47949					
Ti57	Ti	-0.25000	0.00000	-0.25000	O117	O	-0.39527	0.10418	-0.02059					
Ti58	Ti	-0.25000	0.00000	0.25000	O118	O	-0.39814	0.10703	0.47979					
Ti59	Ti	-0.25000	-0.50000	-0.25000	O119	O	-0.39524	-0.39519	-0.02132					
Ti60	Ti	-0.25000	-0.50000	0.25000	O120	O	-0.39553	-0.39506	0.47848					

Table SI8. Atomic coordinates obtained from the DFT calculation for the sample CTG at P = 3.942 GPa.

Atom label	Atom type	*x*	*y*	*z*	Atom label	Atom type	*x*	*y*	*z*	Atom label	Atom type	*x*	*y*	*z*
Ca1	Ca	-0.00500	0.02185	0.12510	Ti61	Ti	0.25000	0.00000	-0.25000	O121	O	-0.10344	-0.10371	0.22951
Ca2	Ca	-0.00452	0.02165	-0.37495	Ti62	Ti	0.25000	0.00000	0.25000	O122	O	-0.10335	-0.10419	-0.26993
Ca3	Ca	-0.00474	-0.47682	0.12438	Ti63	Ti	0.25000	-0.50000	-0.25000	O123	O	-0.10337	0.39628	0.22841
Ca4	Ca	-0.00419	-0.47761	-0.37538	Ti64	Ti	0.25000	-0.50000	0.25000	O124	O	-0.10343	0.39626	-0.27156
Ca5	Ca	0.49579	0.02250	0.12449	O65	O	0.04119	0.23957	0.12497	O125	O	0.39665	-0.10370	0.22839
Ca6	Ca	0.49532	0.02320	-0.37556	O66	O	0.04075	0.23983	-0.37501	O126	O	0.39674	-0.10364	-0.27144
Ca7	Ca	0.49540	-0.47830	0.12505	O67	O	0.03911	-0.25871	0.12255	O127	O	0.39661	0.39578	0.23003
Ca8	Ca	0.49507	-0.47796	-0.37485	O68	O	0.03925	-0.25943	-0.37458	O128	O	0.39664	0.39644	-0.27039
Ca9	Ca	0.00468	-0.02320	-0.12444	O69	O	-0.46073	0.24049	0.12539	O129	O	0.14342	-0.14779	-0.02051
Ca10	Ca	0.00421	-0.02250	0.37551	O70	O	-0.46097	0.24125	-0.37742	O130	O	0.14633	-0.14518	0.47941
Ca11	Ca	0.00493	0.47797	-0.12515	O71	O	-0.45924	-0.26010	0.12499	O131	O	0.14526	0.35546	-0.02166
Ca12	Ca	0.00460	0.47831	0.37495	O72	O	-0.45892	-0.26027	-0.37500	O132	O	0.14594	0.35440	0.47856
Ca13	Ca	-0.49548	-0.02165	-0.12505	O73	O	-0.03903	-0.24125	-0.12258	O133	O	-0.35411	-0.14555	-0.02156
Ca14	Ca	-0.49500	-0.02185	0.37490	O74	O	-0.03927	-0.24049	0.37461	O134	O	-0.35465	-0.14454	0.47839
Ca15	Ca	-0.49581	0.47762	-0.12462	O75	O	-0.04108	0.26027	-0.12500	O135	O	-0.35376	0.35488	-0.02071
Ca16	Ca	-0.49526	0.47683	0.37562	O76	O	-0.04076	0.26011	0.37501	O136	O	-0.35664	0.35227	0.47947
Ca17	Ca	0.24556	0.22736	-0.12465	O77	O	0.45925	-0.23983	-0.12499	O137	O	-0.14656	0.14620	0.22849
Ca18	Ca	0.24534	0.22772	0.37496	O78	O	0.45881	-0.23956	0.37503	O138	O	-0.14635	0.14594	-0.27148
Ca19	Ca	0.24490	-0.27162	-0.12493	O79	O	0.46075	0.25944	-0.12542	O139	O	-0.14667	-0.35369	0.22958
Ca20	Ca	0.24460	-0.27217	0.37509	O80	O	0.46089	0.25872	0.37745	O140	O	-0.14669	-0.35346	-0.27027
Ca21	Ca	-0.25530	0.22785	-0.12497	O81	O	-0.20990	0.01072	-0.12508	O141	O	0.35333	0.14651	0.22966
Ca22	Ca	-0.25509	0.22833	0.37516	O82	O	-0.21053	0.01109	0.37520	O142	O	0.35334	0.14630	-0.27038
Ca23	Ca	-0.25457	-0.27224	-0.12500	O83	O	-0.20957	-0.49085	-0.12504	O143	O	0.35375	-0.35413	0.22848
Ca24	Ca	-0.25444	-0.27259	0.37536	O84	O	-0.21035	-0.49181	0.37521	O144	O	0.35334	-0.35372	-0.27133
Ca25	Ca	-0.24491	-0.22833	0.12484	O85	O	0.28951	0.00818	-0.12479	O145	O	-0.10173	-0.10692	0.02029
Ca26	Ca	-0.24470	-0.22784	-0.37503	O86	O	0.29045	0.00917	0.37494	O146	O	-0.10459	-0.10407	-0.47934
Ca27	Ca	-0.24556	0.27259	0.12464	O87	O	0.28938	-0.48896	-0.12478	O147	O	-0.10432	0.39519	0.02161
Ca28	Ca	-0.24543	0.27225	-0.37500	O88	O	0.29014	-0.48932	0.37495	O148	O	-0.10463	0.39531	-0.47860
Ca29	Ca	0.25466	-0.22771	0.12504	O89	O	0.20955	-0.00916	0.12506	O149	O	0.39537	-0.10466	0.02138
Ca30	Ca	0.25444	-0.22736	-0.37535	O90	O	0.21049	-0.00817	-0.37521	O150	O	0.39585	-0.10464	-0.47843
Ca31	Ca	0.25540	0.27218	0.12491	O91	O	0.20986	0.48932	0.12505	O151	O	0.39538	0.39593	0.02058
Ca32	Ca	0.25510	0.27162	-0.37507	O92	O	0.21062	0.48896	-0.37522	O152	O	0.39833	0.39310	-0.47959
Ge33	Ge	0.00003	-0.25002	-0.00002	O93	O	-0.28947	-0.01108	0.12480	O153	O	0.10327	0.10365	-0.22856
Ti34	Ti	-0.00001	-0.25002	-0.49994	O94	O	-0.29010	-0.01072	-0.37492	O154	O	0.10335	0.10370	0.27161
Ti35	Ti	-0.00002	0.24991	0.00003	O95	O	-0.28965	0.49182	0.12479	O155	O	0.10335	-0.39644	-0.22961
Ti36	Ti	0.00004	0.25003	-0.49995	O96	O	-0.29043	0.49085	-0.37496	O156	O	0.10338	-0.39578	0.26997
Ti37	Ti	-0.50004	-0.25002	-0.00005	O97	O	-0.14535	0.14454	0.02161	O157	O	-0.39665	0.10420	-0.23007
Ti38	Ti	-0.49998	-0.24991	-0.50003	O98	O	-0.14589	0.14555	-0.47844	O158	O	-0.39656	0.10372	0.27049
Ti39	Ti	-0.49999	0.25002	-0.00006	O99	O	-0.14337	-0.35226	0.02053	O159	O	-0.39657	-0.39625	-0.22844
Ge40	Ge	-0.50003	0.25003	-0.49998	O100	O	-0.14624	-0.35487	-0.47929	O160	O	-0.39664	-0.39627	0.27159
Ti41	Ti	0.00013	-0.25036	-0.24888	O101	O	0.35367	0.14519	0.02059					
Ti42	Ti	-0.00014	-0.24969	0.24890	O102	O	0.35658	0.14779	-0.47949					
Ti43	Ti	0.00002	0.24982	-0.24991	O103	O	0.35406	-0.35439	0.02144					
Ti44	Ti	-0.00004	0.25011	0.24993	O104	O	0.35474	-0.35546	-0.47834					
Ti45	Ti	-0.50002	-0.24982	-0.25009	O105	O	0.14666	-0.14629	-0.22962					
Ti46	Ti	-0.49996	-0.25011	0.25007	O106	O	0.14667	-0.14651	0.27034					
Ti47	Ti	-0.50013	0.25036	-0.25111	O107	O	0.14666	0.35372	-0.22867					
Ti48	Ti	-0.49986	0.24970	0.25110	O108	O	0.14625	0.35413	0.27152					
Ti49	Ti	-0.24894	-0.00131	-0.00023	O109	O	-0.35365	-0.14593	-0.22852					
Ti50	Ti	-0.25106	0.00132	-0.49977	O110	O	-0.35344	-0.14620	0.27151					
Ti51	Ti	-0.24870	-0.49895	-0.00022	O111	O	-0.35331	0.35347	-0.22973					
Ti52	Ti	-0.25130	-0.50104	-0.49978	O112	O	-0.35333	0.35370	0.27042					
Ti53	Ti	0.24866	-0.00108	0.00024	O113	O	0.10415	0.10465	-0.02157					
Ti54	Ti	0.25134	0.00108	-0.50024	O114	O	0.10463	0.10466	0.47862					
Ti55	Ti	0.24881	-0.49872	0.00022	O115	O	0.10167	-0.39310	-0.02041					
Ti56	Ti	0.25118	-0.50128	-0.50022	O116	O	0.10462	-0.39592	0.47942					
Ti57	Ti	-0.25000	0.00000	-0.25000	O117	O	-0.39541	0.10408	-0.02066					
Ti58	Ti	-0.25000	0.00000	0.25000	O118	O	-0.39827	0.10693	0.47971					
Ti59	Ti	-0.25000	-0.50000	-0.25000	O119	O	-0.39537	-0.39531	-0.02140					
Ti60	Ti	-0.25000	-0.50000	0.25000	O120	O	-0.39568	-0.39518	0.47839					

Table SI9. Atomic coordinates obtained from the DFT calculation for the sample CTG at P = 5.038 GPa.

Atom label	Atom type	*x*	*y*	*z*	Atom label	Atom type	*x*	*y*	*z*	Atom label	Atom type	*x*	*y*	*z*
Ca1	Ca	-0.00504	0.02200	0.12509	Ti61	Ti	0.25000	0.00000	-0.25000	O121	O	-0.10327	-0.10354	0.22945
Ca2	Ca	-0.00455	0.02178	-0.37494	Ti62	Ti	0.25000	0.00000	0.25000	O122	O	-0.10318	-0.10404	-0.27002
Ca3	Ca	-0.00477	-0.47671	0.12442	Ti63	Ti	0.25000	-0.50000	-0.25000	O123	O	-0.10319	0.39643	0.22831
Ca4	Ca	-0.00419	-0.47749	-0.37539	Ti64	Ti	0.25000	-0.50000	0.25000	O124	O	-0.10325	0.39640	-0.27166
Ca5	Ca	0.49578	0.02262	0.12448	O65	O	0.04144	0.23956	0.12497	O125	O	0.39682	-0.10356	0.22828
Ca6	Ca	0.49531	0.02332	-0.37554	O66	O	0.04095	0.23979	-0.37500	O126	O	0.39689	-0.10350	-0.27155
Ca7	Ca	0.49535	-0.47817	0.12504	O67	O	0.03925	-0.25871	0.12255	O127	O	0.39679	0.39594	0.22993
Ca8	Ca	0.49502	-0.47781	-0.37486	O68	O	0.03941	-0.25942	-0.37456	O128	O	0.39681	0.39660	-0.27046
Ca9	Ca	0.00469	-0.02331	-0.12446	O69	O	-0.46059	0.24051	0.12540	O129	O	0.14354	-0.14791	-0.02058
Ca10	Ca	0.00422	-0.02262	0.37552	O70	O	-0.46084	0.24127	-0.37743	O130	O	0.14649	-0.14528	0.47934
Ca11	Ca	0.00498	0.47781	-0.12514	O71	O	-0.45905	-0.26015	0.12499	O131	O	0.14542	0.35532	-0.02178
Ca12	Ca	0.00465	0.47817	0.37496	O72	O	-0.45868	-0.26028	-0.37500	O132	O	0.14607	0.35428	0.47843
Ca13	Ca	-0.49545	-0.02177	-0.12506	O73	O	-0.03916	-0.24127	-0.12257	O133	O	-0.35397	-0.14567	-0.02167
Ca14	Ca	-0.49495	-0.02200	0.37491	O74	O	-0.03941	-0.24050	0.37460	O134	O	-0.35448	-0.14467	0.47827
Ca15	Ca	-0.49581	0.47749	-0.12461	O75	O	-0.04132	0.26028	-0.12500	O135	O	-0.35359	0.35476	-0.02078
Ca16	Ca	-0.49523	0.47671	0.37558	O76	O	-0.04095	0.26015	0.37501	O136	O	-0.35653	0.35216	0.47941
Ca17	Ca	0.24557	0.22721	-0.12465	O77	O	0.45904	-0.23979	-0.12500	O137	O	-0.14675	0.14636	0.22838
Ca18	Ca	0.24534	0.22757	0.37494	O78	O	0.45856	-0.23955	0.37503	O138	O	-0.14650	0.14606	-0.27159
Ca19	Ca	0.24490	-0.27174	-0.12494	O79	O	0.46059	0.25943	-0.12544	O139	O	-0.14683	-0.35356	0.22953
Ca20	Ca	0.24455	-0.27230	0.37509	O80	O	0.46075	0.25871	0.37745	O140	O	-0.14688	-0.35330	-0.27037
Ca21	Ca	-0.25535	0.22771	-0.12496	O81	O	-0.20971	0.01079	-0.12509	O141	O	0.35312	0.14669	0.22957
Ca22	Ca	-0.25512	0.22822	0.37516	O82	O	-0.21030	0.01113	0.37519	O142	O	0.35317	0.14644	-0.27044
Ca23	Ca	-0.25458	-0.27240	-0.12503	O83	O	-0.20937	-0.49086	-0.12505	O143	O	0.35359	-0.35400	0.22838
Ca24	Ca	-0.25444	-0.27274	0.37537	O84	O	-0.21013	-0.49182	0.37521	O144	O	0.35317	-0.35356	-0.27145
Ca25	Ca	-0.24488	-0.22822	0.12484	O85	O	0.28973	0.00816	-0.12479	O145	O	-0.10160	-0.10682	0.02034
Ca26	Ca	-0.24465	-0.22771	-0.37504	O86	O	0.29063	0.00915	0.37493	O146	O	-0.10447	-0.10393	-0.47926
Ca27	Ca	-0.24557	0.27275	0.12463	O87	O	0.28961	-0.48892	-0.12479	O147	O	-0.10417	0.39530	0.02172
Ca28	Ca	-0.24542	0.27241	-0.37497	O88	O	0.29034	-0.48925	0.37494	O148	O	-0.10446	0.39546	-0.47849
Ca29	Ca	0.25466	-0.22756	0.12506	O89	O	0.20936	-0.00915	0.12507	O149	O	0.39554	-0.10452	0.02149
Ca30	Ca	0.25443	-0.22721	-0.37535	O90	O	0.21027	-0.00816	-0.37521	O150	O	0.39598	-0.10454	-0.47831
Ca31	Ca	0.25545	0.27231	0.12491	O91	O	0.20966	0.48926	0.12506	O151	O	0.39550	0.39606	0.02067
Ca32	Ca	0.25510	0.27174	-0.37506	O92	O	0.21039	0.48892	-0.37521	O152	O	0.39846	0.39321	-0.47954
Ge33	Ge	0.00003	-0.25002	-0.00002	O93	O	-0.28969	-0.01112	0.12481	O153	O	0.10311	0.10351	-0.22845
Ti34	Ti	-0.00001	-0.25002	-0.49994	O94	O	-0.29029	-0.01078	-0.37491	O154	O	0.10318	0.10356	0.27171
Ti35	Ti	-0.00002	0.24993	0.00004	O95	O	-0.28986	0.49183	0.12479	O155	O	0.10319	-0.39659	-0.22954
Ti36	Ti	0.00006	0.25002	-0.49995	O96	O	-0.29063	0.49087	-0.37495	O156	O	0.10321	-0.39593	0.27007
Ti37	Ti	-0.50006	-0.25001	-0.00005	O97	O	-0.14552	0.14468	0.02173	O157	O	-0.39682	0.10405	-0.22997
Ti38	Ti	-0.49998	-0.24992	-0.50004	O98	O	-0.14603	0.14568	-0.47833	O158	O	-0.39673	0.10355	0.27055
Ti39	Ti	-0.50000	0.25003	-0.00006	O99	O	-0.14348	-0.35215	0.02059	O159	O	-0.39675	-0.39639	-0.22834
Ge40	Ge	-0.50004	0.25003	-0.49998	O100	O	-0.14641	-0.35476	-0.47922	O160	O	-0.39681	-0.39642	0.27169
Ti41	Ti	0.00011	-0.25036	-0.24887	O101	O	0.35351	0.14529	0.02066					
Ti42	Ti	-0.00013	-0.24968	0.24889	O102	O	0.35646	0.14792	-0.47942					
Ti43	Ti	0.00001	0.24982	-0.24990	O103	O	0.35393	-0.35428	0.02157					
Ti44	Ti	-0.00004	0.25010	0.24993	O104	O	0.35458	-0.35532	-0.47822					
Ti45	Ti	-0.50001	-0.24982	-0.25010	O105	O	0.14683	-0.14643	-0.22956					
Ti46	Ti	-0.49996	-0.25009	0.25007	O106	O	0.14688	-0.14668	0.27043					
Ti47	Ti	-0.50012	0.25037	-0.25113	O107	O	0.14683	0.35357	-0.22855					
Ti48	Ti	-0.49987	0.24968	0.25111	O108	O	0.14641	0.35400	0.27162					
Ti49	Ti	-0.24891	-0.00133	-0.00024	O109	O	-0.35350	-0.14605	-0.22841					
Ti50	Ti	-0.25109	0.00134	-0.49976	O110	O	-0.35325	-0.14635	0.27162					
Ti51	Ti	-0.24870	-0.49892	-0.00021	O111	O	-0.35312	0.35331	-0.22963					
Ti52	Ti	-0.25130	-0.50107	-0.49979	O112	O	-0.35317	0.35357	0.27047					
Ti53	Ti	0.24864	-0.00110	0.00023	O113	O	0.10402	0.10454	-0.02169					
Ti54	Ti	0.25136	0.00110	-0.50023	O114	O	0.10446	0.10452	0.47851					
Ti55	Ti	0.24878	-0.49870	0.00020	O115	O	0.10154	-0.39321	-0.02046					
Ti56	Ti	0.25121	-0.50129	-0.50020	O116	O	0.10450	-0.39605	0.47933					
Ti57	Ti	-0.25000	0.00000	-0.25000	O117	O	-0.39553	0.10394	-0.02074					
Ti58	Ti	-0.25000	0.00000	0.25000	O118	O	-0.39840	0.10683	0.47966					
Ti59	Ti	-0.25000	-0.50000	-0.25000	O119	O	-0.39554	-0.39545	-0.02151					
Ti60	Ti	-0.25000	-0.50000	0.25000	O120	O	-0.39583	-0.39529	0.47828					

Table SI10. Atomic coordinates obtained from the DFT calculation for the sample CTG at P = 7.679 GPa.

Atom label	Atom type	*x*	*y*	*z*	Atom label	Atom type	*x*	*y*	*z*	Atom label	Atom type	*x*	*y*	*z*
Ca1	Ca	-0.00509	0.02203	0.12504	Ti61	Ti	0.25000	0.00000	-0.25000	O121	O	-0.10293	-0.10319	0.22942
Ca2	Ca	-0.00470	0.02182	-0.37499	Ti62	Ti	0.25000	0.00000	0.25000	O122	O	-0.10290	-0.10387	-0.27017
Ca3	Ca	-0.00467	-0.47680	0.12442	Ti63	Ti	0.25000	-0.50000	-0.25000	O123	O	-0.10293	0.39660	0.22819
Ca4	Ca	-0.00422	-0.47749	-0.37536	Ti64	Ti	0.25000	-0.50000	0.25000	O124	O	-0.10296	0.39660	-0.27178
Ca5	Ca	0.49573	0.02254	0.12460	O65	O	0.04179	0.23973	0.12497	O125	O	0.39703	-0.10337	0.22818
Ca6	Ca	0.49536	0.02322	-0.37555	O66	O	0.04138	0.23992	-0.37500	O126	O	0.39712	-0.10331	-0.27175
Ca7	Ca	0.49521	-0.47813	0.12499	O67	O	0.03935	-0.25860	0.12252	O127	O	0.39708	0.39610	0.22981
Ca8	Ca	0.49492	-0.47787	-0.37494	O68	O	0.03970	-0.25941	-0.37452	O128	O	0.39715	0.39688	-0.27056
Ca9	Ca	0.00464	-0.02321	-0.12445	O69	O	-0.46033	0.24061	0.12546	O129	O	0.14370	-0.14813	-0.02064
Ca10	Ca	0.00427	-0.02253	0.37540	O70	O	-0.46068	0.24141	-0.37748	O130	O	0.14687	-0.14541	0.47919
Ca11	Ca	0.00508	0.47787	-0.12506	O71	O	-0.45862	-0.26006	0.12500	O131	O	0.14578	0.35510	-0.02200
Ca12	Ca	0.00479	0.47814	0.37501	O72	O	-0.45829	-0.26017	-0.37504	O132	O	0.14629	0.35412	0.47818
Ca13	Ca	-0.49530	-0.02182	-0.12501	O73	O	-0.03932	-0.24141	-0.12252	O133	O	-0.35376	-0.14588	-0.02185
Ca14	Ca	-0.49491	-0.02203	0.37496	O74	O	-0.03967	-0.24060	0.37454	O134	O	-0.35417	-0.14487	0.47801
Ca15	Ca	-0.49578	0.47750	-0.12464	O75	O	-0.04171	0.26018	-0.12496	O135	O	-0.35319	0.35461	-0.02087
Ca16	Ca	-0.49533	0.47680	0.37558	O76	O	-0.04138	0.26006	0.37500	O136	O	-0.35632	0.35194	0.47935
Ca17	Ca	0.24574	0.22714	-0.12465	O77	O	0.45862	-0.23991	-0.12500	O137	O	-0.14705	0.14654	0.22823
Ca18	Ca	0.24539	0.22755	0.37502	O78	O	0.45821	-0.23972	0.37503	O138	O	-0.14680	0.14626	-0.27177
Ca19	Ca	0.24480	-0.27165	-0.12497	O79	O	0.46030	0.25941	-0.12548	O139	O	-0.14714	-0.35334	0.22947
Ca20	Ca	0.24443	-0.27221	0.37516	O80	O	0.46065	0.25861	0.37748	O140	O	-0.14718	-0.35308	-0.27050
Ca21	Ca	-0.25553	0.22781	-0.12486	O81	O	-0.20931	0.01082	-0.12509	O141	O	0.35283	0.14691	0.22949
Ca22	Ca	-0.25521	0.22838	0.37506	O82	O	-0.21005	0.01119	0.37521	O142	O	0.35283	0.14671	-0.27048
Ca23	Ca	-0.25457	-0.27246	-0.12497	O83	O	-0.20893	-0.49102	-0.12506	O143	O	0.35325	-0.35376	0.22824
Ca24	Ca	-0.25422	-0.27285	0.37536	O84	O	-0.20977	-0.49203	0.37523	O144	O	0.35292	-0.35339	-0.27170
Ca25	Ca	-0.24479	-0.22837	0.12494	O85	O	0.29014	0.00797	-0.12477	O145	O	-0.10128	-0.10674	0.02048
Ca26	Ca	-0.24447	-0.22781	-0.37514	O86	O	0.29108	0.00899	0.37494	O146	O	-0.10425	-0.10365	-0.47915
Ca27	Ca	-0.24578	0.27285	0.12464	O87	O	0.28988	-0.48886	-0.12481	O147	O	-0.10385	0.39553	0.02197
Ca28	Ca	-0.24543	0.27246	-0.37503	O88	O	0.29070	-0.48921	0.37492	O148	O	-0.10421	0.39565	-0.47832
Ca29	Ca	0.25461	-0.22755	0.12497	O89	O	0.20892	-0.00899	0.12506	O149	O	0.39578	-0.10432	0.02166
Ca30	Ca	0.25426	-0.22714	-0.37535	O90	O	0.20986	-0.00796	-0.37523	O150	O	0.39623	-0.10438	-0.47804
Ca31	Ca	0.25557	0.27221	0.12484	O91	O	0.20930	0.48921	0.12508	O151	O	0.39571	0.39637	0.02082
Ca32	Ca	0.25520	0.27166	-0.37503	O92	O	0.21011	0.48886	-0.37519	O152	O	0.39876	0.39332	-0.47948
Ge33	Ge	0.00001	-0.25001	0.00000	O93	O	-0.28995	-0.01118	0.12479	O153	O	0.10288	0.10331	-0.22825
Ti34	Ti	0.00002	-0.25002	-0.49998	O94	O	-0.29068	-0.01081	-0.37491	O154	O	0.10297	0.10338	0.27182
Ti35	Ti	-0.00001	0.24997	0.00003	O95	O	-0.29023	0.49204	0.12477	O155	O	0.10285	-0.39688	-0.22944
Ti36	Ti	0.00001	0.24999	-0.49999	O96	O	-0.29106	0.49103	-0.37494	O156	O	0.10293	-0.39610	0.27020
Ti37	Ti	-0.50001	-0.24999	-0.00001	O97	O	-0.14583	0.14488	0.02199	O157	O	-0.39709	0.10388	-0.22983
Ti38	Ti	-0.49999	-0.24996	-0.50003	O98	O	-0.14625	0.14588	-0.47815	O158	O	-0.39707	0.10320	0.27058
Ti39	Ti	-0.50002	0.25002	-0.00002	O99	O	-0.14368	-0.35193	0.02064	O159	O	-0.39704	-0.39660	-0.22822
Ge40	Ge	-0.50001	0.25001	-0.50000	O100	O	-0.14681	-0.35460	-0.47913	O160	O	-0.39706	-0.39660	0.27181
Ti41	Ti	-0.00001	-0.25039	-0.24876	O101	O	0.35313	0.14542	0.02081					
Ti42	Ti	0.00001	-0.24961	0.24877	O102	O	0.35630	0.14813	-0.47936					
Ti43	Ti	0.00004	0.24988	-0.24989	O103	O	0.35371	-0.35412	0.02182					
Ti44	Ti	-0.00006	0.25007	0.24989	O104	O	0.35422	-0.35509	-0.47800					
Ti45	Ti	-0.50004	-0.24988	-0.25011	O105	O	0.14717	-0.14670	-0.22951					
Ti46	Ti	-0.49994	-0.25007	0.25011	O106	O	0.14716	-0.14691	0.27051					
Ti47	Ti	-0.49999	0.25040	-0.25124	O107	O	0.14708	0.35339	-0.22830					
Ti48	Ti	-0.50001	0.24962	0.25123	O108	O	0.14675	0.35377	0.27176					
Ti49	Ti	-0.24877	-0.00136	-0.00021	O109	O	-0.35320	-0.14625	-0.22823					
Ti50	Ti	-0.25123	0.00137	-0.49979	O110	O	-0.35296	-0.14653	0.27177					
Ti51	Ti	-0.24862	-0.49876	-0.00013	O111	O	-0.35282	0.35309	-0.22950					
Ti52	Ti	-0.25138	-0.50123	-0.49987	O112	O	-0.35286	0.35334	0.27053					
Ti53	Ti	0.24858	-0.00125	0.00012	O113	O	0.10377	0.10438	-0.02196					
Ti54	Ti	0.25142	0.00125	-0.50012	O114	O	0.10422	0.10433	0.47834					
Ti55	Ti	0.24869	-0.49865	0.00018	O115	O	0.10124	-0.39331	-0.02052					
Ti56	Ti	0.25131	-0.50135	-0.50018	O116	O	0.10429	-0.39636	0.47918					
Ti57	Ti	-0.25000	0.00000	-0.25000	O117	O	-0.39576	0.10366	-0.02084					
Ti58	Ti	-0.25000	0.00000	0.25000	O118	O	-0.39872	0.10675	0.47952					
Ti59	Ti	-0.25000	-0.50000	-0.25000	O119	O	-0.39579	-0.39565	-0.02168					
Ti60	Ti	-0.25000	-0.50000	0.25000	O120	O	-0.39615	-0.39553	0.47803					

Table SI11. Atomic coordinates obtained from the DFT calculation for the sample CTG at P = 10.055 GPa.

Atom label	Atom type	*x*	*y*	*z*
Ca1	Ca	-0.00524	0.02263	0.12504
Ca2	Ca	-0.00473	0.02235	-0.37497
Ca3	Ca	-0.00488	-0.47612	0.12449
Ca4	Ca	-0.00429	-0.47685	-0.37539
Ca5	Ca	0.49566	0.02325	0.12450
Ca6	Ca	0.49520	0.02390	-0.37549
Ca7	Ca	0.49514	-0.47758	0.12499
Ca8	Ca	0.49481	-0.47718	-0.37493
Ca9	Ca	0.00480	-0.02390	-0.12451
Ca10	Ca	0.00434	-0.02325	0.37550
Ca11	Ca	0.00519	0.47718	-0.12507
Ca12	Ca	0.00486	0.47758	0.37501
Ca13	Ca	-0.49527	-0.02235	-0.12503
Ca14	Ca	-0.49476	-0.02263	0.37495
Ca15	Ca	-0.49571	0.47686	-0.12461
Ca16	Ca	-0.49512	0.47613	0.37551
Ca17	Ca	0.24556	0.22654	-0.12462
Ca18	Ca	0.24530	0.22691	0.37491
Ca19	Ca	0.24477	-0.27228	-0.12494
Ca20	Ca	0.24433	-0.27291	0.37514
Ca21	Ca	-0.25559	0.22711	-0.12488
Ca22	Ca	-0.25529	0.22772	0.37514
Ca23	Ca	-0.25464	-0.27309	-0.12507
Ca24	Ca	-0.25442	-0.27343	0.37540
Ca25	Ca	-0.24471	-0.22771	0.12486
Ca26	Ca	-0.24441	-0.22711	-0.37512
Ca27	Ca	-0.24558	0.27344	0.12460
Ca28	Ca	-0.24536	0.27310	-0.37492
Ca29	Ca	0.25470	-0.22691	0.12509
Ca30	Ca	0.25444	-0.22653	-0.37538
Ca31	Ca	0.25567	0.27292	0.12486
Ca32	Ca	0.25523	0.27228	-0.37506
Ge33	Ge	0.00004	-0.25002	-0.00001
Ti34	Ti	0.00002	-0.25004	-0.49995
Ti35	Ti	-0.00002	0.24995	0.00005
Ti36	Ti	0.00006	0.25000	-0.49997
Ti37	Ti	-0.50006	-0.24999	-0.00003
Ti38	Ti	-0.49998	-0.24994	-0.50005
Ti39	Ti	-0.50002	0.25004	-0.00005
Ge40	Ge	-0.50004	0.25002	-0.49999
Ti41	Ti	0.00004	-0.25038	-0.24878
Ti42	Ti	-0.00006	-0.24963	0.24880
Ti43	Ti	0.00001	0.24984	-0.24989
Ti44	Ti	-0.00007	0.25006	0.24991
Ti45	Ti	-0.50001	-0.24984	-0.25011
Ti46	Ti	-0.49993	-0.25005	0.25009
Ti47	Ti	-0.50004	0.25039	-0.25122
Ti48	Ti	-0.49994	0.24964	0.25120
Ti49	Ti	-0.24880	-0.00140	-0.00023
Ti50	Ti	-0.25120	0.00141	-0.49977
Ti51	Ti	-0.24867	-0.49879	-0.00017
Ti52	Ti	-0.25133	-0.50120	-0.49983
Ti53	Ti	0.24858	-0.00122	0.00018
Ti54	Ti	0.25142	0.00122	-0.50018
Ti55	Ti	0.24867	-0.49863	0.00017
Ti56	Ti	0.25133	-0.50137	-0.50017
Ti57	Ti	-0.25000	0.00000	-0.25000
Ti58	Ti	-0.25000	0.00000	0.25000
Ti59	Ti	-0.25000	-0.50000	-0.25000
Ti60	Ti	-0.25000	-0.50000	0.25000
Ti61	Ti	0.25000	0.00000	-0.25000
Ti62	Ti	0.25000	0.00000	0.25000
Ti63	Ti	0.25000	-0.50000	-0.25000
Ti64	Ti	0.25000	-0.50000	0.25000
O65	O	0.04240	0.23951	0.12497
O66	O	0.04180	0.23970	-0.37500
O67	O	0.03982	-0.25861	0.12251
O68	O	0.04013	-0.25937	-0.37450
O69	O	-0.45991	0.24060	0.12546
O70	O	-0.46027	0.24140	-0.37747
O71	O	-0.45822	-0.26027	0.12499
O72	O	-0.45775	-0.26031	-0.37501
O73	O	-0.03973	-0.24140	-0.12253
O74	O	-0.04009	-0.24060	0.37454
O75	O	-0.04225	0.26032	-0.12499
O76	O	-0.04178	0.26027	0.37501
O77	O	0.45820	-0.23970	-0.12500
O78	O	0.45760	-0.23951	0.37503
O79	O	0.45987	0.25937	-0.12550
O80	O	0.46018	0.25861	0.37749
O81	O	-0.20887	0.01095	-0.12510
O82	O	-0.20946	0.01125	0.37518
O83	O	-0.20851	-0.49092	-0.12508
O84	O	-0.20927	-0.49191	0.37523
O85	O	0.29058	0.00808	-0.12477
O86	O	0.29149	0.00911	0.37492
O87	O	0.29045	-0.48881	-0.12481
O88	O	0.29117	-0.48910	0.37494
O89	O	0.20851	-0.00911	0.12508
O90	O	0.20942	-0.00807	-0.37523
O91	O	0.20883	0.48910	0.12506
O92	O	0.20955	0.48881	-0.37519
O93	O	-0.29054	-0.01125	0.12482
O94	O	-0.29113	-0.01094	-0.37490
O95	O	-0.29073	0.49191	0.12477
O96	O	-0.29149	0.49093	-0.37492
O97	O	-0.14621	0.14522	0.02221
O98	O	-0.14666	0.14626	-0.47790
O99	O	-0.14399	-0.35164	0.02085
O100	O	-0.14714	-0.35426	-0.47889
O101	O	0.35279	0.14577	0.02100
O102	O	0.35593	0.14845	-0.47914
O103	O	0.35329	-0.35373	0.02203
O104	O	0.35389	-0.35475	-0.47776
O105	O	0.14754	-0.14702	-0.22926
O106	O	0.14765	-0.14734	0.27080
O107	O	0.14756	0.35295	-0.22811
O108	O	0.14710	0.35343	0.27201
O109	O	-0.35282	-0.14660	-0.22800
O110	O	-0.35250	-0.14695	0.27205
O111	O	-0.35237	0.35267	-0.22924
O112	O	-0.35248	0.35301	0.27078
O113	O	0.10339	0.10403	-0.02215
O114	O	0.10378	0.10394	0.47809
O115	O	0.10092	-0.39365	-0.02072
O116	O	0.10393	-0.39665	0.47898
O117	O	-0.39609	0.10335	-0.02107
O118	O	-0.39904	0.10641	0.47938
O119	O	-0.39623	-0.39604	-0.02194
O120	O	-0.39649	-0.39582	0.47782
O121	O	-0.10258	-0.10289	0.22916
O122	O	-0.10248	-0.10346	-0.27040
O123	O	-0.10251	0.39699	0.22791
O124	O	-0.10252	0.39699	-0.27208
O125	O	0.39751	-0.10296	0.22787
O126	O	0.39756	-0.10293	-0.27197
O127	O	0.39752	0.39652	0.22957
O128	O	0.39752	0.39724	-0.27077
O129	O	0.14407	-0.14844	-0.02086
O130	O	0.14720	-0.14576	0.47900
O131	O	0.14611	0.35475	-0.02224
O132	O	0.14671	0.35374	0.47797
O133	O	-0.35334	-0.14625	-0.02210
O134	O	-0.35379	-0.14522	0.47779
O135	O	-0.35286	0.35427	-0.02111
O136	O	-0.35601	0.35165	0.47915
O137	O	-0.14750	0.14696	0.22795
O138	O	-0.14718	0.14660	-0.27200
O139	O	-0.14752	-0.35300	0.22922
O140	O	-0.14763	-0.35267	-0.27076
O141	O	0.35235	0.14735	0.22920
O142	O	0.35246	0.14703	-0.27074
O143	O	0.35290	-0.35343	0.22799
O144	O	0.35244	-0.35295	-0.27189
O145	O	-0.10096	-0.10640	0.02062
O146	O	-0.10391	-0.10334	-0.47893
O147	O	-0.10351	0.39582	0.02218
O148	O	-0.10377	0.39605	-0.47806
O149	O	0.39622	-0.10393	0.02191
O150	O	0.39661	-0.10403	-0.47785
O151	O	0.39606	0.39665	0.02102
O152	O	0.39908	0.39365	-0.47928
O153	O	0.10244	0.10294	-0.22803
O154	O	0.10249	0.10296	0.27213
O155	O	0.10248	-0.39724	-0.22923
O156	O	0.10248	-0.39651	0.27043
O157	O	-0.39752	0.10347	-0.22960
O158	O	-0.39742	0.10289	0.27084
O159	O	-0.39748	-0.39698	-0.22792
O160	O	-0.39749	-0.39699	0.27209

Table SI12. Atomic coordinates obtained from the DFT calculation for the sample CTG at *P* = 15.079 GPa.

Atom label	Atom type	*x*	*y*	*z*	Atom label	Atom type	*x*	*y*	*z*	Atom label	Atom type	*x*	*y*	*z*
Ca1	Ca	-0.00543	0.02322	0.12501	Ti61	Ti	0.25000	0.00000	-0.25000	O121	O	-0.10195	-0.10234	0.22885
Ca2	Ca	-0.00491	0.02291	-0.37500	Ti62	Ti	0.25000	0.00000	0.25000	O122	O	-0.10185	-0.10293	-0.27072
Ca3	Ca	-0.00501	-0.47553	0.12453	Ti63	Ti	0.25000	-0.50000	-0.25000	O123	O	-0.10190	0.39750	0.22754
Ca4	Ca	-0.00441	-0.47620	-0.37540	Ti64	Ti	0.25000	-0.50000	0.25000	O124	O	-0.10187	0.39752	-0.27246
Ca5	Ca	0.49552	0.02391	0.12450	O65	O	0.04325	0.23949	0.12498	O125	O	0.39816	-0.10242	0.22748
Ca6	Ca	0.49506	0.02451	-0.37545	O66	O	0.04258	0.23967	-0.37500	O126	O	0.39817	-0.10241	-0.27234
Ca7	Ca	0.49496	-0.47702	0.12495	O67	O	0.04038	-0.25845	0.12246	O127	O	0.39816	0.39704	0.22925
Ca8	Ca	0.49462	-0.47658	-0.37497	O68	O	0.04081	-0.25927	-0.37448	O128	O	0.39816	0.39780	-0.27109
Ca9	Ca	0.00494	-0.02450	-0.12455	O69	O	-0.45925	0.24072	0.12548	O129	O	0.14455	-0.14892	-0.02112
Ca10	Ca	0.00448	-0.02391	0.37550	O70	O	-0.45972	0.24157	-0.37751	O130	O	0.14783	-0.14623	0.47869
Ca11	Ca	0.00538	0.47658	-0.12503	O71	O	-0.45745	-0.26032	0.12499	O131	O	0.14671	0.35425	-0.02264
Ca12	Ca	0.00504	0.47703	0.37505	O72	O	-0.45694	-0.26032	-0.37503	O132	O	0.14733	0.35320	0.47759
Ca13	Ca	-0.49509	-0.02291	-0.12500	O73	O	-0.04028	-0.24156	-0.12249	O133	O	-0.35272	-0.14680	-0.02248
Ca14	Ca	-0.49457	-0.02322	0.37499	O74	O	-0.04075	-0.24072	0.37452	O134	O	-0.35318	-0.14571	0.47736
Ca15	Ca	-0.49559	0.47621	-0.12460	O75	O	-0.04306	0.26033	-0.12497	O135	O	-0.35224	0.35380	-0.02141
Ca16	Ca	-0.49499	0.47553	0.37547	O76	O	-0.04255	0.26032	0.37501	O136	O	-0.35554	0.35119	0.47890
Ca17	Ca	0.24550	0.22590	-0.12461	O77	O	0.45742	-0.23966	-0.12500	O137	O	-0.14815	0.14750	0.22758
Ca18	Ca	0.24520	0.22630	0.37488	O78	O	0.45675	-0.23948	0.37502	O138	O	-0.14780	0.14711	-0.27237
Ca19	Ca	0.24463	-0.27283	-0.12494	O79	O	0.45919	0.25928	-0.12552	O139	O	-0.14814	-0.35249	0.22890
Ca20	Ca	0.24412	-0.27352	0.37519	O80	O	0.45962	0.25845	0.37754	O140	O	-0.14830	-0.35211	-0.27110
Ca21	Ca	-0.25579	0.22652	-0.12483	O81	O	-0.20807	0.01100	-0.12510	O141	O	0.35168	0.14791	0.22886
Ca22	Ca	-0.25544	0.22719	0.37515	O82	O	-0.20869	0.01130	0.37519	O142	O	0.35184	0.14755	-0.27105
Ca23	Ca	-0.25473	-0.27372	-0.12511	O83	O	-0.20772	-0.49098	-0.12509	O143	O	0.35230	-0.35291	0.22764
Ca24	Ca	-0.25445	-0.27407	0.37541	O84	O	-0.20850	-0.49201	0.37523	O144	O	0.35179	-0.35240	-0.27226
Ca25	Ca	-0.24456	-0.22718	0.12485	O85	O	0.29134	0.00798	-0.12476	O145	O	-0.10038	-0.10601	0.02087
Ca26	Ca	-0.24421	-0.22651	-0.37517	O86	O	0.29229	0.00908	0.37490	O146	O	-0.10336	-0.10283	-0.47863
Ca27	Ca	-0.24555	0.27407	0.12459	O87	O	0.29121	-0.48878	-0.12482	O147	O	-0.10290	0.39632	0.02259
Ca28	Ca	-0.24528	0.27373	-0.37489	O88	O	0.29196	-0.48907	0.37494	O148	O	-0.10315	0.39657	-0.47769
Ca29	Ca	0.25480	-0.22630	0.12512	O89	O	0.20771	-0.00907	0.12510	O149	O	0.39684	-0.10341	0.02229
Ca30	Ca	0.25450	-0.22590	-0.37539	O90	O	0.20865	-0.00797	-0.37524	O150	O	0.39722	-0.10352	-0.47745
Ca31	Ca	0.25588	0.27352	0.12481	O91	O	0.20804	0.48907	0.12506	O151	O	0.39660	0.39717	0.02132
Ca32	Ca	0.25537	0.27283	-0.37506	O92	O	0.20879	0.48878	-0.37518	O152	O	0.39967	0.39405	-0.47903
Ge33	Ge	0.00004	-0.25002	-0.00001	O93	O	-0.29131	-0.01130	0.12481	O153	O	0.10183	0.10242	-0.22766
Ti34	Ti	0.00003	-0.25003	-0.49995	O94	O	-0.29193	-0.01099	-0.37490	O154	O	0.10184	0.10242	0.27252
Ti35	Ti	-0.00002	0.24995	0.00006	O95	O	-0.29150	0.49202	0.12477	O155	O	0.10183	-0.39780	-0.22891
Ti36	Ti	0.00005	0.25000	-0.49997	O96	O	-0.29228	0.49098	-0.37491	O156	O	0.10184	-0.39704	0.27075
Ti37	Ti	-0.50005	-0.24999	-0.00003	O97	O	-0.14682	0.14572	0.02263	O157	O	-0.39815	0.10294	-0.22928
Ti38	Ti	-0.49998	-0.24994	-0.50006	O98	O	-0.14729	0.14680	-0.47752	O158	O	-0.39805	0.10234	0.27115
Ti39	Ti	-0.50003	0.25004	-0.00005	O99	O	-0.14446	-0.35118	0.02110	O159	O	-0.39813	-0.39751	-0.22754
Ge40	Ge	-0.50004	0.25002	-0.49999	O100	O	-0.14776	-0.35379	-0.47859	O160	O	-0.39810	-0.39749	0.27246
Ti41	Ti	-0.00001	-0.25039	-0.24872	O101	O	0.35217	0.14624	0.02131					
Ti42	Ti	-0.00001	-0.24962	0.24874	O102	O	0.35545	0.14892	-0.47888					
Ti43	Ti	0.00001	0.24986	-0.24989	O103	O	0.35267	-0.35320	0.02242					
Ti44	Ti	-0.00008	0.25003	0.24990	O104	O	0.35329	-0.35424	-0.47736					
Ti45	Ti	-0.50001	-0.24985	-0.25011	O105	O	0.14816	-0.14754	-0.22895					
Ti46	Ti	-0.49992	-0.25003	0.25010	O106	O	0.14832	-0.14790	0.27114					
Ti47	Ti	-0.49999	0.25040	-0.25128	O107	O	0.14821	0.35240	-0.22774					
Ti48	Ti	-0.49999	0.24962	0.25126	O108	O	0.14770	0.35291	0.27236					
Ti49	Ti	-0.24871	-0.00145	-0.00021	O109	O	-0.35220	-0.14710	-0.22763					
Ti50	Ti	-0.25129	0.00145	-0.49979	O110	O	-0.35185	-0.14749	0.27242					
Ti51	Ti	-0.24863	-0.49870	-0.00014	O111	O	-0.35171	0.35212	-0.22890					
Ti52	Ti	-0.25137	-0.50129	-0.49986	O112	O	-0.35186	0.35250	0.27110					
Ti53	Ti	0.24854	-0.00130	0.00016	O113	O	0.10278	0.10353	-0.02255					
Ti54	Ti	0.25145	0.00131	-0.50016	O114	O	0.10316	0.10342	0.47771					
Ti55	Ti	0.24859	-0.49858	0.00015	O115	O	0.10033	-0.39405	-0.02097					
Ti56	Ti	0.25141	-0.50142	-0.50015	O116	O	0.10340	-0.39717	0.47868					
Ti57	Ti	-0.25000	0.00000	-0.25000	O117	O	-0.39664	0.10284	-0.02137					
Ti58	Ti	-0.25000	0.00000	0.25000	O118	O	-0.39962	0.10602	0.47913					
Ti59	Ti	-0.25000	-0.50000	-0.25000	O119	O	-0.39685	-0.39657	-0.02231					
Ti60	Ti	-0.25000	-0.50000	0.25000	O120	O	-0.39710	-0.39632	0.47741					

Table SI13. Atomic coordinates obtained from the DFT calculation for the sample CTG at P = 20.081 GPa.

Atom label	Atom type	*x*	*y*	*z*
Ca1	Ca	-0.00567	0.02381	0.12497
Ca2	Ca	-0.00518	0.02349	-0.37503
Ca3	Ca	-0.00518	-0.47493	0.12454
Ca4	Ca	-0.00461	-0.47555	-0.37539
Ca5	Ca	0.49532	0.02455	0.12453
Ca6	Ca	0.49487	0.02510	-0.37544
Ca7	Ca	0.49470	-0.47644	0.12492
Ca8	Ca	0.49437	-0.47601	-0.37501
Ca9	Ca	0.00513	-0.02510	-0.12456
Ca10	Ca	0.00468	-0.02454	0.37547
Ca11	Ca	0.00563	0.47601	-0.12499
Ca12	Ca	0.00530	0.47644	0.37508
Ca13	Ca	-0.49483	-0.02349	-0.12497
Ca14	Ca	-0.49433	-0.02380	0.37503
Ca15	Ca	-0.49540	0.47555	-0.12461
Ca16	Ca	-0.49482	0.47494	0.37546
Ca17	Ca	0.24541	0.22523	-0.12460
Ca18	Ca	0.24504	0.22569	0.37488
Ca19	Ca	0.24439	-0.27335	-0.12495
Ca20	Ca	0.24383	-0.27409	0.37525
Ca21	Ca	-0.25608	0.22595	-0.12477
Ca22	Ca	-0.25566	0.22669	0.37512
Ca23	Ca	-0.25488	-0.27434	-0.12512
Ca24	Ca	-0.25450	-0.27474	0.37541
Ca25	Ca	-0.24434	-0.22669	0.12488
Ca26	Ca	-0.24392	-0.22594	-0.37523
Ca27	Ca	-0.24550	0.27475	0.12459
Ca28	Ca	-0.24512	0.27435	-0.37488
Ca29	Ca	0.25496	-0.22569	0.12512
Ca30	Ca	0.25459	-0.22523	-0.37540
Ca31	Ca	0.25617	0.27409	0.12475
Ca32	Ca	0.25560	0.27335	-0.37505
Ge33	Ge	0.00004	-0.25002	-0.00001
Ti34	Ti	0.00004	-0.25003	-0.49996
Ti35	Ti	-0.00002	0.24995	0.00006
Ti36	Ti	0.00003	0.24999	-0.49998
Ti37	Ti	-0.50004	-0.24999	-0.00002
Ti38	Ti	-0.49998	-0.24994	-0.50006
Ti39	Ti	-0.50004	0.25004	-0.00004
Ge40	Ge	-0.50004	0.25002	-0.49999
Ti41	Ti	-0.00010	-0.25042	-0.24865
Ti42	Ti	0.00008	-0.24957	0.24866
Ti43	Ti	0.00002	0.24988	-0.24987
Ti44	Ti	-0.00010	0.25000	0.24987
Ti45	Ti	-0.50002	-0.24987	-0.25013
Ti46	Ti	-0.49990	-0.24999	0.25013
Ti47	Ti	-0.49990	0.25043	-0.25135
Ti48	Ti	-0.50008	0.24957	0.25134
Ti49	Ti	-0.24861	-0.00149	-0.00019
Ti50	Ti	-0.25139	0.00149	-0.49981
Ti51	Ti	-0.24858	-0.49859	-0.00010
Ti52	Ti	-0.25142	-0.50140	-0.49990
Ti53	Ti	0.24850	-0.00142	0.00012
Ti54	Ti	0.25149	0.00143	-0.50012
Ti55	Ti	0.24851	-0.49854	0.00012
Ti56	Ti	0.25149	-0.50146	-0.50011
Ti57	Ti	-0.25000	0.00000	-0.25000
Ti58	Ti	-0.25000	0.00000	0.25000
Ti59	Ti	-0.25000	-0.50000	-0.25000
Ti60	Ti	-0.25000	-0.50000	0.25000
Ti61	Ti	0.25000	0.00000	-0.25000
Ti62	Ti	0.25000	0.00000	0.25000
Ti63	Ti	0.25000	-0.50000	-0.25000
Ti64	Ti	0.25000	-0.50000	0.25000
O65	O	0.04398	0.23950	0.12497
O66	O	0.04328	0.23968	-0.37500
O67	O	0.04080	-0.25827	0.12242
O68	O	0.04139	-0.25917	-0.37445
O69	O	-0.45870	0.24086	0.12551
O70	O	-0.45928	0.24176	-0.37756
O71	O	-0.45673	-0.26031	0.12500
O72	O	-0.45621	-0.26030	-0.37504
O73	O	-0.04072	-0.24176	-0.12244
O74	O	-0.04130	-0.24085	0.37449
O75	O	-0.04379	0.26031	-0.12496
O76	O	-0.04327	0.26032	0.37500
O77	O	0.45672	-0.23967	-0.12500
O78	O	0.45602	-0.23949	0.37503
O79	O	0.45861	0.25917	-0.12555
O80	O	0.45920	0.25827	0.37758
O81	O	-0.20737	0.01104	-0.12510
O82	O	-0.20805	0.01138	0.37520
O83	O	-0.20700	-0.49108	-0.12510
O84	O	-0.20783	-0.49219	0.37524
O85	O	0.29200	0.00781	-0.12476
O86	O	0.29302	0.00898	0.37489
O87	O	0.29185	-0.48872	-0.12483
O88	O	0.29265	-0.48903	0.37494
O89	O	0.20698	-0.00898	0.12511
O90	O	0.20800	-0.00781	-0.37524
O91	O	0.20734	0.48903	0.12506
O92	O	0.20815	0.48872	-0.37517
O93	O	-0.29195	-0.01137	0.12480
O94	O	-0.29263	-0.01103	-0.37490
O95	O	-0.29217	0.49219	0.12476
O96	O	-0.29300	0.49109	-0.37490
O97	O	-0.14743	0.14620	0.02302
O98	O	-0.14789	0.14731	-0.47717
O99	O	-0.14494	-0.35073	0.02129
O100	O	-0.14839	-0.35335	-0.47833
O101	O	0.35153	0.14667	0.02158
O102	O	0.35498	0.14938	-0.47869
O103	O	0.35207	-0.35270	0.02277
O104	O	0.35267	-0.35376	-0.47700
O105	O	0.14879	-0.14806	-0.22872
O106	O	0.14898	-0.14843	0.27140
O107	O	0.14883	0.35190	-0.22739
O108	O	0.14831	0.35240	0.27267
O109	O	-0.35159	-0.14759	-0.22731
O110	O	-0.35120	-0.14798	0.27275
O111	O	-0.35105	0.35160	-0.22862
O112	O	-0.35125	0.35200	0.27133
O113	O	0.10218	0.10305	-0.02291
O114	O	0.10256	0.10293	0.47739
O115	O	0.09974	-0.39442	-0.02117
O116	O	0.10286	-0.39769	0.47842
O117	O	-0.39718	0.10232	-0.02161
O118	O	-0.40021	0.10566	0.47892
O119	O	-0.39745	-0.39706	-0.02264
O120	O	-0.39771	-0.39680	0.47704
O121	O	-0.10130	-0.10177	0.22864
O122	O	-0.10124	-0.10245	-0.27099
O123	O	-0.10130	0.39797	0.22723
O124	O	-0.10124	0.39802	-0.27276
O125	O	0.39878	-0.10191	0.22718
O126	O	0.39877	-0.10193	-0.27267
O127	O	0.39878	0.39754	0.22899
O128	O	0.39882	0.39835	-0.27133
O129	O	0.14502	-0.14938	-0.02131
O130	O	0.14847	-0.14667	0.47842
O131	O	0.14733	0.35376	-0.02300
O132	O	0.14793	0.35270	0.47723
O133	O	-0.35211	-0.14730	-0.02283
O134	O	-0.35257	-0.14619	0.47698
O135	O	-0.35161	0.35335	-0.02167
O136	O	-0.35506	0.35074	0.47871
O137	O	-0.14880	0.14799	0.22725
O138	O	-0.14841	0.14760	-0.27269
O139	O	-0.14875	-0.35199	0.22867
O140	O	-0.14895	-0.35159	-0.27138
O141	O	0.35102	0.14844	0.22860
O142	O	0.35121	0.14807	-0.27128
O143	O	0.35169	-0.35240	0.22733
O144	O	0.35117	-0.35189	-0.27261
O145	O	-0.09979	-0.10565	0.02108
O146	O	-0.10282	-0.10232	-0.47839
O147	O	-0.10229	0.39680	0.02296
O148	O	-0.10255	0.39706	-0.47736
O149	O	0.39744	-0.10292	0.02261
O150	O	0.39782	-0.10305	-0.47708
O151	O	0.39714	0.39769	0.02158
O152	O	0.40026	0.39443	-0.47883
O153	O	0.10123	0.10193	-0.22733
O154	O	0.10122	0.10191	0.27282
O155	O	0.10117	-0.39835	-0.22867
O156	O	0.10122	-0.39753	0.27101
O157	O	-0.39876	0.10246	-0.22901
O158	O	-0.39870	0.10177	0.27136
O159	O	-0.39876	-0.39802	-0.22724
O160	O	-0.39870	-0.39796	0.27277

Table SI14. Atomic coordinates obtained from the DFT calculation for the sample CTG at P = 25.141 GPa.

Atom label	Atom type	*x*	*y*	*z*	Atom label	Atom type	*x*	*y*	*z*	Atom label	Atom type	*x*	*y*	*z*
Ca1	Ca	-0.00594	0.02439	0.12494	Ti61	Ti	0.25000	0.00000	-0.25000	O121	O	-0.10060	-0.10117	0.22847
Ca2	Ca	-0.00549	0.02407	-0.37506	Ti62	Ti	0.25000	0.00000	0.25000	O122	O	-0.10062	-0.10195	-0.27120
Ca3	Ca	-0.00538	-0.47435	0.12455	Ti63	Ti	0.25000	-0.50000	-0.25000	O123	O	-0.10067	0.39846	0.22694
Ca4	Ca	-0.00484	-0.47487	-0.37538	Ti64	Ti	0.25000	-0.50000	0.25000	O124	O	-0.10060	0.39853	-0.27302
Ca5	Ca	0.49509	0.02519	0.12456	O65	O	0.04464	0.23951	0.12495	O125	O	0.39942	-0.10139	0.22693
Ca6	Ca	0.49465	0.02568	-0.37542	O66	O	0.04396	0.23972	-0.37499	O126	O	0.39939	-0.10142	-0.27299
Ca7	Ca	0.49440	-0.47586	0.12489	O67	O	0.04116	-0.25805	0.12241	O127	O	0.39942	0.39805	0.22879
Ca8	Ca	0.49408	-0.47545	-0.37504	O68	O	0.04188	-0.25905	-0.37443	O128	O	0.39952	0.39892	-0.27153
Ca9	Ca	0.00535	-0.02568	-0.12458	O69	O	-0.45822	0.24099	0.12554	O129	O	0.14555	-0.14985	-0.02145
Ca10	Ca	0.00491	-0.02519	0.37544	O70	O	-0.45890	0.24199	-0.37758	O130	O	0.14914	-0.14711	0.47819
Ca11	Ca	0.00592	0.47545	-0.12496	O71	O	-0.45603	-0.26026	0.12501	O131	O	0.14798	0.35327	-0.02335
Ca12	Ca	0.00560	0.47586	0.37511	O72	O	-0.45552	-0.26028	-0.37506	O132	O	0.14855	0.35219	0.47690
Ca13	Ca	-0.49451	-0.02407	-0.12494	O73	O	-0.04110	-0.24199	-0.12242	O133	O	-0.35149	-0.14782	-0.02315
Ca14	Ca	-0.49406	-0.02438	0.37506	O74	O	-0.04178	-0.24099	0.37446	O134	O	-0.35195	-0.14668	0.47662
Ca15	Ca	-0.49516	0.47488	-0.12462	O75	O	-0.04448	0.26028	-0.12494	O135	O	-0.35095	0.35290	-0.02187
Ca16	Ca	-0.49462	0.47435	0.37545	O76	O	-0.04397	0.26027	0.37499	O136	O	-0.35453	0.35027	0.47857
Ca17	Ca	0.24529	0.22454	-0.12460	O77	O	0.45604	-0.23971	-0.12501	O137	O	-0.14947	0.14850	0.22696
Ca18	Ca	0.24482	0.22508	0.37488	O78	O	0.45536	-0.23951	0.37505	O138	O	-0.14904	0.14811	-0.27298
Ca19	Ca	0.24411	-0.27385	-0.12498	O79	O	0.45812	0.25906	-0.12557	O139	O	-0.14940	-0.35145	0.22849
Ca20	Ca	0.24349	-0.27465	0.37533	O80	O	0.45884	0.25806	0.37759	O140	O	-0.14963	-0.35108	-0.27162
Ca21	Ca	-0.25643	0.22539	-0.12471	O81	O	-0.20671	0.01107	-0.12510	O141	O	0.35034	0.14896	0.22839
Ca22	Ca	-0.25592	0.22621	0.37508	O82	O	-0.20750	0.01149	0.37521	O142	O	0.35056	0.14861	-0.27145
Ca23	Ca	-0.25511	-0.27495	-0.12512	O83	O	-0.20633	-0.49121	-0.12510	O143	O	0.35104	-0.35187	0.22705
Ca24	Ca	-0.25459	-0.27544	0.37541	O84	O	-0.20724	-0.49241	0.37524	O144	O	0.35052	-0.35138	-0.27293
Ca25	Ca	-0.24408	-0.22621	0.12492	O85	O	0.29259	0.00758	-0.12476	O145	O	-0.09920	-0.10526	0.02125
Ca26	Ca	-0.24357	-0.22538	-0.37529	O86	O	0.29369	0.00886	0.37489	O146	O	-0.10223	-0.10179	-0.47820
Ca27	Ca	-0.24541	0.27545	0.12459	O87	O	0.29242	-0.48862	-0.12484	O147	O	-0.10164	0.39731	0.02330
Ca28	Ca	-0.24489	0.27495	-0.37488	O88	O	0.29331	-0.48899	0.37494	O148	O	-0.10193	0.39756	-0.47707
Ca29	Ca	0.25518	-0.22508	0.12512	O89	O	0.20631	-0.00885	0.12511	O149	O	0.39806	-0.10244	0.02291
Ca30	Ca	0.25471	-0.22454	-0.37540	O90	O	0.20741	-0.00757	-0.37524	O150	O	0.39846	-0.10254	-0.47674
Ca31	Ca	0.25651	0.27466	0.12467	O91	O	0.20668	0.48899	0.12506	O151	O	0.39772	0.39823	0.02178
Ca32	Ca	0.25589	0.27386	-0.37502	O92	O	0.20758	0.48862	-0.37516	O152	O	0.40085	0.39484	-0.47866
Ge33	Ge	0.00003	-0.25002	-0.00001	O93	O	-0.29250	-0.01149	0.12479	O153	O	0.10061	0.10143	-0.22701
Ti34	Ti	0.00004	-0.25002	-0.49997	O94	O	-0.29329	-0.01106	-0.37490	O154	O	0.10058	0.10140	0.27307
Ti35	Ti	-0.00002	0.24995	0.00005	O95	O	-0.29275	0.49242	0.12476	O155	O	0.10048	-0.39891	-0.22848
Ti36	Ti	0.00002	0.24999	-0.49999	O96	O	-0.29367	0.49122	-0.37490	O156	O	0.10058	-0.39805	0.27121
Ti37	Ti	-0.50002	-0.24998	-0.00001	O97	O	-0.14805	0.14669	0.02338	O157	O	-0.39938	0.10196	-0.22880
Ti38	Ti	-0.49998	-0.24994	-0.50005	O98	O	-0.14851	0.14783	-0.47685	O158	O	-0.39940	0.10117	0.27153
Ti39	Ti	-0.50004	0.25003	-0.00003	O99	O	-0.14548	-0.35026	0.02143	O159	O	-0.39940	-0.39853	-0.22698
Ge40	Ge	-0.50003	0.25002	-0.49999	O100	O	-0.14905	-0.35289	-0.47813	O160	O	-0.39934	-0.39846	0.27306
Ti41	Ti	-0.00020	-0.25045	-0.24859	O101	O	0.35086	0.14711	0.02181					
Ti42	Ti	0.00019	-0.24953	0.24859	O102	O	0.35445	0.14985	-0.47855					
Ti43	Ti	0.00003	0.24990	-0.24985	O103	O	0.35145	-0.35218	0.02310					
Ti44	Ti	-0.00011	0.24999	0.24984	O104	O	0.35202	-0.35326	-0.47665					
Ti45	Ti	-0.50003	-0.24990	-0.25015	O105	O	0.14944	-0.14861	-0.22855					
Ti46	Ti	-0.49989	-0.24998	0.25016	O106	O	0.14966	-0.14896	0.27161					
Ti47	Ti	-0.49980	0.25046	-0.25141	O107	O	0.14948	0.35139	-0.22707					
Ti48	Ti	-0.50019	0.24953	0.25141	O108	O	0.14896	0.35187	0.27295					
Ti49	Ti	-0.24852	-0.00151	-0.00016	O109	O	-0.35096	-0.14810	-0.22702					
Ti50	Ti	-0.25148	0.00152	-0.49984	O110	O	-0.35053	-0.14849	0.27304					
Ti51	Ti	-0.24854	-0.49850	-0.00007	O111	O	-0.35038	0.35109	-0.22838					
Ti52	Ti	-0.25146	-0.50149	-0.49993	O112	O	-0.35060	0.35146	0.27151					
Ti53	Ti	0.24848	-0.00152	0.00008	O113	O	0.10154	0.10255	-0.02326					
Ti54	Ti	0.25152	0.00152	-0.50008	O114	O	0.10194	0.10244	0.47710					
Ti55	Ti	0.24844	-0.49851	0.00010	O115	O	0.09915	-0.39483	-0.02134					
Ti56	Ti	0.25156	-0.50148	-0.50010	O116	O	0.10228	-0.39822	0.47822					
Ti57	Ti	-0.25000	0.00000	-0.25000	O117	O	-0.39777	0.10180	-0.02180					
Ti58	Ti	-0.25000	0.00000	0.25000	O118	O	-0.40080	0.10526	0.47875					
Ti59	Ti	-0.25000	-0.50000	-0.25000	O119	O	-0.39807	-0.39755	-0.02293					
Ti60	Ti	-0.25000	-0.50000	0.25000	O120	O	-0.39836	-0.39730	0.47669					

Table S15. Unit-cell parameters (*a*, *b*, *c*, and *V*) and selected bond lengths and angles at room conditions for $CaTiO_3$ orthorhombic perovskites from the literature and those for the CTG sample investigated here, derived from X-ray diffraction experiments.

a (Å)	*b* Å)	*c* (Å)	*V* (Å^3)	*B*–O1 (Å)	*B*–O2 (Å)	*B*–O2' (Å)	⟨*B*–O⟩ (Å)	*V*(BO_6) (Å^3)	ECoN	*B*–O1–*B* (°)	*B*–O2–*B* (°)	Reference
5.3829(3)	7.6453(4)	5.4458(3)	224.12(4)	1.952(2)	1.953(2)	1.963(2)	1.956(2)	9.98	5.999	156.3	155.8	5
5.3796(1)	5.4423(3)	7.6401(5)	223.68(1)	1.9503(3)	1.9551(8)	1.9587(8)	1.9547	9.96	5.999	156.67(8)	155.71(5)	6
5.3785(2)	5.4419(2)	7.6400(3)	223.62(3)	1.939(1)	1.950(1)	1.957(1)	1.952	9.86	5.997	156.8	158.2	7
5.380(1)	5.440(1)	7.639(1)	223.56(7)	1.950	1.957	1.957	1.955	9.95	5.999	156.8	155.6	8
5.378(1)	5.444(1)	7.637(3)	223.59	1.951	1.958	1.958	1.956	9.97	5.999	156.3	155.4	9
5.3814(1)	5.4418(1)	7.6409(2)	223.76	1.947	1.953	1.968	1.956	9.97	5.996	157.8	154.9	10
5.3827(5)	5.4537(5)	7.6551(9)	224.72	1.952	1.959	1.964	1.958	10.01	5.999	157.2	155.2	11
5.3799(5)	5.4411(5)	7.6411(8)	223.67	1.950	1.950	1.960	1.955	9.95	5.999	156.7	155.7	12
5.4043(8)	5.4224(7)	7.6510(12)	224.19	1.946(1)	1.951(3)	1.960(3)	1952(2)	9.90(4)	5.998	158.7	156.3	13
5.378(7)	5.439(7)	7.638(1)	223.42	1.954	1.960	1.969	1.961	10.05	5.998	152.0	155.4	14
5.38052(7)	5.44251(6)	7.64096(9)	223.754(8)	1.951	1.954	1.958	1.954	9.95	6.000	156.7	156.0	15
5.38159(6)	5.44281(7)	7.64207(10)	223.843(5)	1.949(1)	1.953(3)	1.961(3)	1.954(2)	9.953(2)	5.999	157.1	155.8	16
5.3789(2)	5.4361(2)	7.6388(3)	223.36	1.951	1.953	1.961	1.955	9.96	5.999	156.0	155.6	17
5.3810(3)	5.4445(4)	7.6484(2)	224.07	1.952	1.956	1.961	1.953	9.98	5.999	156.9	155.5	18
5.38095(2)	5.43710(2)	7.64208(5)	223.58	1.951	1.956	1.958	1.955	9.96	6.000	156.5	155.6	19
5.3709(2)	5.4280(2)	7.6268(3)	222.35(2)	1.946	1.949	1.958	1.951	9.90	5.999	156.8	155.6	20
5.3828	5.4460	7.6391	223.94	1.950	1.956	1.960	1.955	9.97	5.999	156.7	155.7	21
5.3786(2)	5.4322(2)	7.6397(3)	223.21	1.948(1)	1.950(1)	1.964(3)	1.954	9.94	5.997	157.0(1)	155.3(3)	22
5.38176(22)	5.44403(21)	7.6425(7)	223.913(25)	1.9513(10)	1.9585(24)	1.9587(25)	1.957	9.981(19)	5.999	156.39(28)	155.32(26)	23
5.38079(12)	5.44273(12)	7.64138(16)	223.787(8)	1.948(3)	1.963(3)	1.9548(10)	1.955	9.96	5.998	155.5(2)	156.3(2)	24
5.38097(2)	5.43780(2)	7.64012(3)	223.555(1)	1.9494(4)	1.9517(11)	1.9556(11)	1.9522(9)	9.92	6.000	156.94(10)	156.44(7)	*This work*

Table S3 references

(5) Koopmans, H. J. A.; Van De Velde, G. M. H.;, Gellings, P. J. Powder neutron diffraction study of the perovskites $CaTiO_3$ and $CaZrO_3$. *Acta Crystallogr. C* **1983**, *39*(10), 1323-1325.

(6) Sasaki, S.; Prewitt, C. T.; Bass, J. D.; Schulze, W. A. Orthorhombic perovskite $CaTiO_3$ and $CdTiO_3$: structure and space group. *Acta Crystallogr. C* **1987**, *43*(9), 1668-1674.

(7) Liu, X.; Liebermann, R. C. X-ray powder diffraction study of $CaTiO_3$ perovskite at high temperatures. *Phys. Chem. Miner.* **1993**, *20*(3), 171-175.

(8) Beran, A.; Libowitzky, E.; Armbruster, T. A single-crystal infrared spectroscopic and X-ray-diffraction study of untwinned San Benito perovskite containing OH groups. *Can. Mineral.* **1996**, *34*(4), 803-809.

(9) Arakcheeva, A. V.; Lubman, G. U.; Pushcharovskii, D. Y.; Gekimyants, V. M.; Popov, V. A. Crystal structure and microtwinning of natural orthorhombic perovskite $CaTiO_3$. *Crystallogr. Rep.* **1997**, *42*(1), 46-54.

(10) Chakhmouradian, A. R.; Mitchell, R. H. A structural study of the perovskite series $CaTi_{1-2x}Fe_xNb_xO_3$. *J. Solid State Chem.* **1998**, *138*(2), 272-277.

(11) Ranjan, R.; Pandey, D.; Siruguri, V.; Krishna, P. S. R.; Paranjpe, S. K. Novel structural features and phase transition behaviour of $(Sr_{1-x}Ca_x)TiO_3$: I. Neutron diffraction study. *J. Phys.-Condens. Mat.* **1999**, *11*(10), 2233.

(12) Dunyushkina, L. A.; Gorbunov, V. A. Effect of Crystal Structure on the Electrical Properties of $CaTi_{1-x}Fe_xO_{3-\delta}$. *Inorg. Mater.* **2001**, *37*(11), 1165-1169.

(13) Yamanaka, T.; Hirai, N.; Komatsu, Y. Structure change of $Ca_{1-x}Sr_xTiO_3$ perovskite with composition and pressure. *Am. Mineral.* **2002**, *87*(8-9), 1183-1189.

(14) Chandra, A.; Pandey, D. Evolution of crystallographic phases in the system $(Pb_{1-x}Ca_x)TiO_3$: A Rietveld study. *J. Mater. Res.* **2003**, *18*(2), 407-414.

(15) Vashook, V.; Vasylechko, L.; Knapp, M.; Ullmann, H.; Guth, U. Lanthanum doped calcium titanates: synthesis, crystal structure, thermal expansion and transport properties. *J. Alloy. Compd.* **2003**, *354*(1-2), 13-23.

(16) Mitchell, R. H.; Liferovich, R. P. A structural study of the perovskite series $Ca_{1-x}Na_xTi_{1-x}Ta_xO_3$. *J. Solid State Chem.* **2004**, *177*(12), 4420-4427.

(17) Ali, R.;, Yashima, M. Space group and crystal structure of the perovskite $CaTiO_3$ from 296 to 1720 K. *J. Solid State Chem.* **2005**, *178*(9), 2867-2872.

(18) Tsubota, M.; Iga, F.; Uchihira, K.; Nakano, T.; Kura, S.; Takabatake, T.; Kodama, S.; Nakao, H.; Murakami, Y. Coupling between Orbital and Lattice Degrees of Freedom in $Y_{1-x}Ca_xTiO_3$ ($0< x \leq 0.75$): A Resonant X-ray Scattering Study. *J. Phys. Soc. Jpn.* **2005**, *74*(12), 3259-3266.

(19) Knight, K. S. Parameterization of the crystal structures of centrosymmetric zone-boundary-tilted perovskites: an analysis in terms of symmetry-adapted basis-vectors of the cubic aristotype phase. *Can. Mineral.* **2009**, *47*(2), 381-400.

(20) Yashima, M.; Ali, R. Structural phase transition and octahedral tilting in the calcium titanate perovskite $CaTiO_3$. *Solid State Ionics* **2009**, *180*(2-3), 120-126.

(21) Yang, H.; Ohishi, Y.; Kurosaki, K.; Muta, H.; Yamanaka, S. Thermomechanical properties of calcium series perovskite-type oxides. *J. Alloy. Compd.* **2010**, *504*(1), 201-204.

(22) Shahzad, K.; Khan, M. N.; Bashir, J. Synchrotron X-Ray Diffraction Studies of Pure and A-and B-Site Cation Doped $CaTiO_3$. *Ferroelectrics* **2011**, *414*(1), 162-169.

(23) Zhao, J.; Ross, N. L.; Wang, D.; Angel, R. J. High-pressure crystal structure of elastically isotropic $CaTiO_3$ perovskite under hydrostatic and non-hydrostatic conditions. *J. Phys.-Condens. Mat.* **2011**, *23*(45), 455401.

(24) Takani, N.; Yamane, H. Structure analysis of $CaTi_{1-x}Sn_xO_3$ (x= 0.0-1.0) solid solutions. *Powder Diffr.* **2014**, *29*(3), 254.
